# Spatiotemporal patterns in urban nutrient and suspended sediment loads and stream response to watershed management implementation

**DOI:** 10.1007/s10661-025-13917-7

**Published:** 2025-04-01

**Authors:** Aaron J. Porter

**Affiliations:** Virginia-West Virginia Water Science Center, US Geological Survey, 1730 East Parham Road, Richmond, VA 23228 USA

**Keywords:** Sediment, Nutrients, Urban, Chesapeake Bay, Management practices, Flow-normalization, Flow-adjustment

## Abstract

In recent years, local governments have invested heavily in management practices to reduce nutrient and sediment loads. These practices provide localities with nutrient and sediment regulatory reduction credits; however, their effects on water quality are poorly understood at the watershed scale. Long-term watershed-scale monitoring is essential for assessing progress toward water-quality goals, yet it has historically been lacking in urban watersheds. Since 2007, Fairfax County, Virginia, has partnered with the US Geological Survey to monitor and evaluate water-quality conditions in 20 small urban streams. This study assessed nutrient and suspended sediment loads, trends in concentration, and trends in load. Trends in load are affected by streamflow-induced variability that must be removed through a process called “flow-normalization;” however, existing methods have neither been applied to small urban watersheds nor to loads computed on a sub-daily timestep. In this study, four such methods also were assessed, and an adaptation of the weighted regressions on time, discharge, and season approach was found to be most effective. Loads, concentrations, and trends in load were spatially and temporally variable. Differences were attributed to physical watershed features such as geology, soils, and channel geomorphology, as well as urban sources such as turfgrass fertilization and septic infrastructure. Most notably, flow-normalized suspended sediment, nitrogen, and phosphorus loads decreased in two watersheds with completed stream restorations and increased in those with few implemented practices.

**Supplementary Information:**

The online version contains supplementary material available at 10.1007/s10661-025-13917-7.

## Introduction

Urban and suburban lands account for a relatively small proportion of land area across the USA but contain 82% of the nation’s population (US Census Bureau, 2020). These lands are projected to nearly triple from 2009 to 2060 (Terando et al., 2014), and this expansion is expected to negatively impact small urban streams (Van Metre et al., 2019). Decades of research have documented the impact of urbanization on watershed hydrology and stream health (Bhaskar et al., 2016; MacKenzie et al., 2022; Paul & Meyer, 2001; Wahl et al., 1997; Walsh et al., 2005). The term “urban stream syndrome” describes a cascade of processes that cyclically undermine urban streams (Walsh et al., 2005). Land development alters watershed hydrology leading to a change in stream hydraulics. These forces modify stream geomorphology, degrade water quality, and negatively impact aquatic communities.

Management practices are a widely accepted tool for restoring watersheds and streams from nonpoint source pollution; however, the performance of these practices typically is evaluated through short-term studies, and stream response is rarely assessed at the watershed scale—the scale at which regulations are implemented. Expected water quality improvements based on regulatory models often fail to align with monitored results (Scientific and Technical Advisory Committee, 2023). This contradiction suggests that the efficiency at which these practices reduce contaminants remains uncertain and that actual reductions in load may differ from those credited (Ator et al., 2020; Kleinman et al., 2022; Lintern et al., 2020; Liu et al., 2017; Osmond et al., 2012). In the Chesapeake Bay watershed, USA, regulatory models have been used to claim large reductions in nitrogen and phosphorus and to conclude that the sediment total maximum daily load (TMDL) has been met (Scientific and Technical Advisory Committee, 2023). However, monitoring studies suggest that current reductions will not meet these Chesapeake Bay TMDL targets, and the health of the estuary has not improved as anticipated (Chesapeake Bay Program, 2018; Kleinman et al., 2019, Chesapeake Bay Program, 2023a).

Local monitoring data are needed to establish connections between management practices and water quality improvements and to track progress towards regulatory goals (Berger et al., 2024). The efficiency of these practices varies due to local factors such as climate, geology, and land use; therefore, regional regulatory models can be improved by utilizing data from a spatially representative collection of local monitoring studies (Berger et al., 2024). The data these studies can provide should facilitate better alignment between modeled and monitored changes in water quality and support federal, state, and local managers to implement future management strategies more effectively and efficiently.

Repeated measures of load at fixed locations year-over-year (> 10 years) generate the data needed to assess trends (Chanat et al., 2016). These data provide the basis for making direct comparisons between credited reductions and measured change. Establishing links between water quality trends and management practice implementation require a long-term commitment to monitoring post-implementation. Although regulatory credits typically are granted upon completion of a management practice, actual stream response may not be immediate (Webber et al., 2023). Lag times between practice implementation and stream response may result from legacy accumulation and vary by contaminant based on delivery pathway (Van Meter & Basu, 2017; Lintern et al., 2020).

How a stream responds to one or more management practices is affected by the source, mobilization, and delivery characteristics of the contaminant of interest (Granger et al., 2010). For example, sources of nutrients can occur naturally or be introduced to the watershed through urban activities such as lawn fertilization or wastewater leakages. Mobilization occurs when these constituents become detached from their source through processes such as erosion, desorption, or mineralization. Delivery is the connectivity of the source to the stream by surface or subsurface pathways. Management practices are designed to improve water quality by mitigating one or more of these factors. Mitigating excess nutrients and sediment in urban streams typically focuses on reducing sources, limiting mobilization, and disrupting delivery, making a clear understanding of these processes essential for effective management (Ma et al., 2021).

Since 2007, Fairfax County, Virginia, has partnered with the US Geological Survey (USGS) to operate a water-resources monitoring program. The program seeks to establish baseline characterizations of water quality and streamflow conditions in county streams, determine sediment and nutrient loadings, and evaluate relations between changes in water quality and management practice implementations. The county has implemented stream and outfall restorations, bioretention, permeable pavement, infiltration practices, vegetated roofing, constructed wetlands, regenerative stormwater conveyances, dry swales, rainwater harvesting, and the construction of new and maintenance of existing detention ponds (Fairfax County 2023). Understanding the efficacy of these actions is needed because substantial investments (approximately $25 million per year) are made by Fairfax County annually to satisfy Chesapeake Bay and local TMDL requirements. This monitoring program provides the data needed to evaluate current progress and inform future management decisions (Fairfax County, 2023, 2024).

The study presented herein aims to evaluate current conditions in Fairfax County streams and determine how those conditions have changed by analyzing a uniquely comprehensive suite of water quality and streamflow data collected over a 10–15-year period in 20 monitored streams. The objectives of this study were to (1) describe changes in flow-normalized (FN) total nitrogen (TN), total phosphorus (TP), and suspended sediment (SS) concentrations in 20 Fairfax County streams using samples collected each month, typically representing baseflow conditions; (2) calculate network-wide trends in concentration by leveraging data collected at all 20 stations; (3) compute TN, TP, and SS loads and yields (area-normalized load) and trends in FN load in 5 Fairfax County streams; and (4) compare existing and novel methods for removing streamflow-induced variability in load, referred to as flow-normalization.

## Data and methods

### Study area

**Fig. 1:**
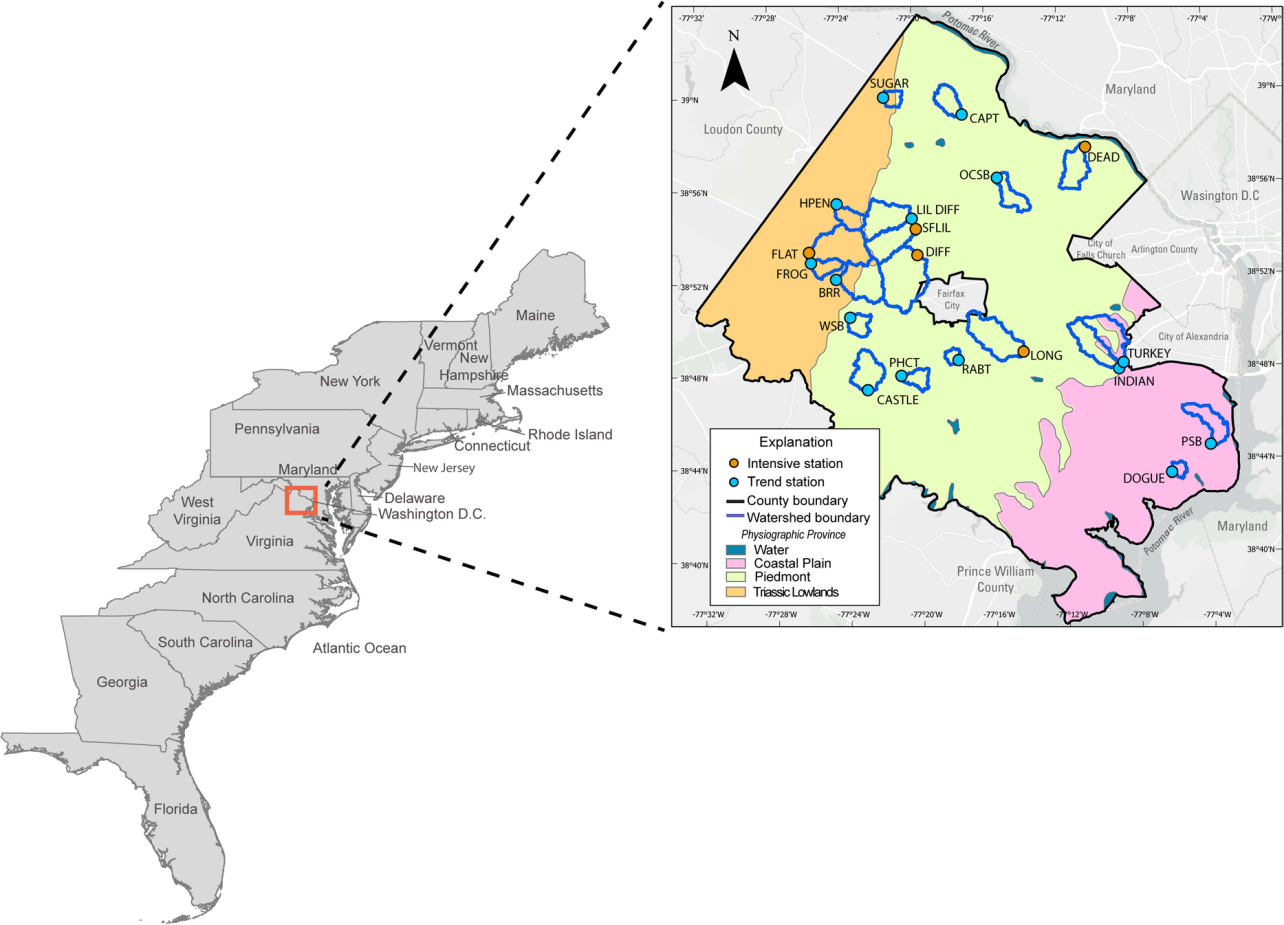
Location of study area along the east coast of the United States in Fairfax County, Virginia. Inset map shows locations of the 20 monitoring stations and watershed boundaries (Table 1). Physiographic provinces from Fenneman, 1938. Light Gray Canvas base map: Esri, TomTom, Garmin, FAO, NOAA, USGS, © OpenStreetMap contributors, and the GIS User Community

The monitoring network, consisting of 14 stations representing small mixed-use urban and suburban watersheds, was established in late 2007; six additional stations were installed in late 2013. The 20-station network is in Fairfax County, Virginia, USA (Table 1; Fig. 1). Fairfax County is a 1032-km^2^ county in northern Virginia and is the most populous jurisdiction in the Commonwealth of Virginia with an estimated population of 1,138,331 as of 2022 that has grown by 12.7% since stream monitoring began (US Census Bureau, 2023a). Development in the study watersheds is primarily commercial and residential single-family homes. Some form of urbanization, such as the development of impervious surfaces, housing units, and public works infrastructure, occurred in all study watersheds during the study period (Webber et al., 2023). Impervious land cover increased by an average of 2.1% from 2008 to 2018 in the study watersheds (US Census Bureau, 2023b; Webber et al., 2023). As of 2018, the county’s land cover was primarily turf grass (37%), forest (30%), and impervious surfaces (25%) (Chesapeake Bay Program, 2023b). The 20 study watersheds range in size from about 1 to 14 km^2^, and total impervious cover from 7.2 to 52.5%. In this study, impervious cover is calculated as the sum of the Chesapeake Bay Programs four related general land use categories (roads, impervious structures, impervious other, and tree canopy over impervious).

**Table 1:** Description of the 20 study watersheds. Physiographic provinces are based on Fenneman (1938)

USGS station ID	Short name	Station type	Watershed area, km^2^	Impervious cover, %	Monitoring period, yrs	Dominant physiographic province
01646305	DEAD	Intensive	5.3	35.7	14	Piedmont
01645704	DIFF	Intensive	14.2	32.2	15	Piedmont
01656903	FLAT	Intensive	10.9	33.4	15	Triassic
01654500	LONG	Intensive	9.6	29.1	10	Piedmont
01645762	SFLIL	Intensive	7.0	15.8	15	Piedmont
0165694286	BRR	Trend	8.8	48.3	15	Piedmont
01645940	CAPT	Trend	3.6	14.8	15	Piedmont
01657394	CASTLE	Trend	5.7	7.2	15	Piedmont
01653844	DOGUE	Trend	1.1	34.1	10	Coastal Plain
0165690673	FROG	Trend	2.6	35.7	15	Triassic
0164425950	HPEN	Trend	3.1	28.2	10	Triassic
01652789	INDIAN	Trend	6.3	38.1	15	Piedmont
01645745	LIL DIFF	Trend	7.7	13.9	15	Piedmont
01645844	OCSB	Trend	3.8	52.5	15	Piedmont
01657322	PHCT	Trend	2.5	21.5	15	Piedmont
01653717	PSB	Trend	4.9	32.4	15	Coastal Plain
01655305	RABT	Trend	1.5	33.9	10	Piedmont
01644343	SUGAR	Trend	1.7	21.1	10	Triassic
01652860	TURKEY	Trend	6.7	34.8	15	Coastal Plain
01657100	WSB	Trend	2.5	25.4	10	Piedmont

Fairfax County sits atop three distinct geologic terranes: sedimentary rocks in the Triassic Lowlands, Crystalline rock in the Piedmont, and a mixture of sands and clays in the Coastal Plain (Froelich & Zenone, 1985a). These terranes are known to affect streamflow and water quality (Jastram, 2014; Porter et al., 2020; Webber et al., 2023). For example, in the Triassic Lowlands, soils are shallower and streams have higher dissolved-solids concentrations predominantly consisting of calcium bicarbonate.

Since 2009, the Fairfax County has invested $176 million on a suite of management practices that include stream restoration, outfall restoration, bioretention, constructed wetlands, dry swales, filtering practices, detention ponds, permeable pavement, infiltration practices, and vegetated roofs (Fairfax County, 2017; Fairfax County 2023; S. Curtis, 2024, Fairfax County, written communication). These practices treat a combined 250 km^2^ throughout Fairfax County; 89 km^2^ of which are impervious (Fairfax County, 2017; Fairfax County 2023; S. Curtis, 2024 personal communication). Fairfax County receives total nitrogen (TN), total phosphorus (TP), and total suspended solids (TSS; similar to suspended sediment). Chesapeake Bay TMDL credit reductions of approximately 26,000 kg, 7500 kg, and 1200 mt, respectively, for these implemented practices (Fairfax County, 2017; Fairfax County 2023; S. Curtis, 2024 personal communication). Urban stream restorations account for 86% of land area treated and 80%, 87%, and 75% of TN, TP, and TSS credits, respectively.

Practices were implemented in 13 of the 20 study watersheds to varying degrees (Table S1). Watersheds with the greatest percent area treated were FLAT, INDIAN, and DEAD where a combined 3275 linear meters of stream were restored. In the study watersheds, Fairfax County implemented practices that treat a combined 33 km^2^ (30% of the combined land area), 12.8 of which are designated as impervious, and received reduction credits for 3800 kg of TN, 1000 kg of TP, and 350 mt of total suspended solids.

### Study design

The monitoring network consists of two types of monitoring stations characterized by the frequency of data collection; five stations are termed “intensive” and 15 “trend” (Fig. 1). At the five intensive monitoring stations, instrumentation is operated to continuously collect 15-min interval measurements of stage, turbidity, specific conductance, water temperature, dissolved oxygen, and pH. Stage measurements, collected every 5 min, are used to compute a continuous timeseries of streamflow following standard USGS procedures (Rantz, 1982; Sauer & Turnipseed, 2010; US Geological Survey, 2014). At all 20 stations, a water quality sample is collected each month and analyzed for a suite of constituents (Table S2; US Geological Survey, 2006). At the intensive stations, automated samplers are used to collect additional storm targeted samples (approximately 30/yr). Storm samples are collected across the full range of hydrologic conditions, seasons, and years of the study. Data collected at the intensive stations support the computation of load and trend in load, whereas data from all stations support computation of trend in concentration.

### Data sources

All continuous and discrete data were retrieved from the National Water Information System website (NWIS) using the USGS station IDs listed in Table 1 (US Geological Survey, 2023). Monitoring stations have various lengths of record. Records date back to 2008, 2009, or 2013, depending on the station. Years in this study represent water years (WY), defined as October 1 through September 30, and named for the year in which the period ends. Additional datasets not available on NWIS, such as load models, load input files, and baseflow separations are provided in a Porter, 2025. Predictor data were obtained from Webber et al. (2022).

### Surrogate regression models

Water quality in urban streams has received increased focus in recent years, and a growing body of research is utilizing continuous monitoring to reduce uncertainty in load estimates (Viviano, et al., 2014; Rode et al., 2022; Morel et al., 2020; Porter et al., 2020; Aulenbach et al., 2022; Porter, 2022; Aulenbach et al., 2023; Miller et al., 2023). The hydrologic characteristics of small, heavily urbanized watersheds are uniquely capable of generating considerable contaminant loads episodically and over short periods of time. Measures such as streamflow, turbidity, specific conductance, pH, or water temperature that can be collected continuously can be used as surrogates to estimate concentration when sampling data are unavailable (Jastram et al., 2010; Densmore et al., 2016; Schilling et al., 2017). This modeling approach, referred to as “surrogate regression,” uses multiple linear regression to relate discretely collected concentrations to one or more continuously measured parameters that may be correlated (Porter et al., 2020; Robertson et al., 2018; Stone et al., 2024). Additional variance may be explained by incorporating parameters for time, season, or hydrologic condition (Porter, 2022). Concentrations below the detection limit can introduce first-order bias to model coefficients (Cohn, 1988). Adjusted maximum likelihood estimation (AMLE; Cohn, 1988; Cohn et al., 1992) is used to eliminate this bias. The accuracy of loads calculated with this “surrogate” approach is an improvement over methods that rely solely on periodically collected samples (Jastram et al., 2009; Robertson et al., 2018; Leigh et al., 2019).

In this study, a station-specific model was developed for SS, TN, TP, and nutrient subspecies at each of the five intensively monitored stations (Porter, 2025). Models were calibrated using concentration data obtained from samples collected across the range of observed hydrologic conditions, seasons, and full duration of the study and paired surrogate parameters collected continuously, every 15 min. Models were selected to meet all assumptions of linear regression. The explanatory power of each model was maximized while minimizing systemic errors (bias) and random errors (variance) using a suite of criteria detailed in Helsel et al. (2020) including (1) Nash–Sutcliffe index (Nash & Sutcliffe, 1970), (2) partial-load ratio (Stenback et al., 2011), (3) Mallows’ Cp (Mallows, 1973), and (4) analysis of model residuals. Multi-collinearity between predictors was avoided by centering parameters and excluding terms with a variance inflation factor greater than 10. Data were log-transformed to meet the assumption of normality of residuals. Calibrated surrogate models were then used to compute a high-frequency (15 min) timeseries of concentration that was subsequently converted to a load by multiplying each value by streamflow. Loads were computed in R v. 4.3.2 (R Core Team, 2023) using the rloadest package (Lorenz et al., 2015). Serial correlation was handled by the rloadest package using methods detailed in Cohn (2005). Load was converted to yield, area-normalized load, to allow for spatial comparisons. For seasonal comparisons, season was defined as spring (March through May), summer (June through August), fall (September through November), and winter (December through February). Average annual yield of TN, TP, and SS are compared to the Chesapeake Assessment Scenario Tool (CAST) 2008 and 2022 model scenarios for Fairfax County. CAST is the watershed model used by the Chesapeake Bay Program to estimate the effects of changes in land use, management actions, point source, and atmospheric deposition on long-term loads of nitrogen, phosphorus, and sediment delivered to the tidal waters of the Chesapeake Bay (Chesapeake Bay Program, 2020). CAST also is the tool used by Chesapeake Bay Program partners to track progress of efforts to meet the Chesapeake Bay TMDL.

### Flow normalization

A growing number of urban stream monitoring stations utilizing the surrogate regression modeling approach have been continuously operated for over a decade, leading to greater interest in evaluating trends in pollutant loads (US Geological Survey, 2023). The measurement of contaminant load is informative for TMDL development or to understand drivers of biological impairment; however, it is less useful for assessing progress towards a reduction target because loads are strongly related to streamflow, which is in turn driven by short-term randomness and long-term patterns in precipitation (Hirsch et al., 2010). A trend suggesting a reduction in load may result from a sequence of dry years rather than a change in source or transport, which can lead to the incorrect assumption that water-quality improvements have been achieved. To understand how changes in the watershed affect water quality, the influence of streamflow-induced variability must be reduced before computing a trend.

### Trend in flow-normalized concentration

Trends in flow-normalized concentration were computed at all 20 stations with a nonparametric Seasonal Mann–Kendall test in R v. 4.3.2 (R Core Team, 2023) using the restrend package (v. 0.4.2; Lorenz, 2017). The Seasonal Kendall test accounts for seasonality by running the Mann–Kendall test on each month separately and then combining the results (Helsel et al., 2020; Hirsch et al., 1982). This test controls for streamflow-induced variability by first performing a nonparametric regression of concentration and streamflow. The trend test is then conducted on the resulting residuals. A censored local regression method was used to obtain the residuals (Loader, 2013) when a dataset contained greater than 10% censored values (values less than the laboratory detection limit).

### Trend in flow-normalized load

Flow-normalization methods have been proposed to control for streamflow induced variability in load; however, these approaches are (1) best suited for large watersheds (Robertson et al., 2018; Zhang & Hirsch, 2019) or (2) assume the relation between water quality and streamflow is linear and constant over time, such as with discharge-weighting or time-weighting techniques (Rowland et al., 2021). An approach to control for streamflow-induced variability in surrogate-derived loads is needed.

Four flow-normalization methods were assessed, Weighted Regressions on Time, Discharge, and Season (WRTDS), “WRTDS-Surrogate” (WRTDS-S), Locally Estimated Scatterplot Smoothing “LOESS-adjustment,” and “streamflow-averaging to provide insight for this and future studies investigating trends in load in small watersheds. Methods were evaluated based on accuracy of load prediction, flow-normalization efficiency, and trend result. The accuracy of load estimates was examined by regressing annual surrogate-regression model results with non-flow-normalized WRTDS and WRTDS-S loads and comparing the *R*^2^ and root-mean-square error. For this evaluation, the surrogate-modeled load is considered the “true” load. Although this is not actually true, surrogate regression models are considered the most accurate means of estimating concentration in these settings (Jastram et al., 2010; Robertson et al., 2018), and this assumption provides a means to evaluate the other methods. Streamflow-averaging and LOESS-adjustment are strictly FN methods, so these were excluded from the comparison. Flow-normalization, how efficiently each method removed hydrologically driven interannual variability in load, was evaluated by first standardizing each annual timeseries value to a scale from 0 to 1 with the following equation:

1 $${N}_{i}=\frac{{L}_{i}-{L}_{\text{min}}}{{L}_{\text{max}}-{L}_{\text{min}}}$$

where *N*_*i*_ is the standardized annual load in the *i*th year,

*L*_*i*_ is the annual value in the *i*th year,

*L*_min_ is the minimum annual load in the timeseries, and.

*L*_max_ is the maximum annual load in the timeseries.

Variance (*V*) was then calculated for each standardized timeseries by calculating the sum of squared differences between each year with the following equation:

2 $$V=\sum {\left({N}_{i}-{N}_{i-1}\right)}^{2}$$

Mean variance was computed from the five stations and used to compare methods. The amount of variance removed was determined by subtracting mean variance from the variance in the raw surrogate-derived load. Flow-normalized trend results are compared on the basis of the magnitude (% change), direction (increasing or decreasing), and statistical significance (probability or likelihood of a trend) of the result.

#### WRTDS

Weighted regressions on rime, discharge, and season (WRTDS) uses inputs of mean daily streamflow and discrete samples collected from a range of hydrologic conditions, seasons, and years to estimate concentration, load, FN load, and trend in FN load. A unique weighted regression is developed for each day of the non-linear, time varying relations between time, discharge, and season (Hirsch et al., 2010). WRTDS is a statistically robust and well-cited approach for assessing water quality trends and is the method used to compute flow-normalized trends in load at the 123 Chesapeake Bay non-tidal network (NTN) stations. Loads and trends calculated at the NTN stations serve as calibrations points for the Chesapeake Bay watershed model; therefore, use of this method here allows for direct comparison of results. Trends were computed in R v. 4.3.2 using the EGRET package (v. 3.0.9, Hirsch et al., 2015b).

##### WRTDS-surrogate

The WRTDS-S approach, developed by this study, is an adaptation of WRTDS that combines daily estimates of surrogate-regression model derived concentrations with the standard WRTDS methodology for computing FN-load and trend. The surrogate model provides an estimation of concentration every 15 min, which are averaged to compute a mean daily value. These estimates are used in place of periodically collected calibration data, thus mimicking an ideal study design whereby a sample is collected on each day.

##### LOESS-adjustment

Flow adjustment was performed by regressing total monthly load, computed with the surrogate regression model, against total monthly streamflow. A nonparametric local regression smoothing algorithm (commonly referred to as a LOESS curve) was used to fit this relation. Residuals were calculated by subtracting the modeled load from the LOESS curve (Cleveland and Devlin, 1988). The Seasonal Mann–Kendall test in R v. 4.3.2 (R Core Team, 2023) using the restrend package (v. 0.4.2; Lorenz, 2017). and then retransformed to original units. The relation between residuals and time represents the trend in load that is not explained by variations in streamflow.

##### Streamflow-averaging

For each station and constituent, a timeseries of concentration was computed with the surrogate regression model. To compute a load, each 15-min unit value of concentration is traditionally multiplied by the corresponding measured streamflow value. Instead, measured streamflow was replaced with a normalized value. Normalization was achieved by calculating the average streamflow value that occurred at each time of day and day of year over the trend period (Mayo & Leib, 2012). For example, the streamflow value that occurred on January 1st, 2018, at 12:00 AM was replaced with the average of all streamflow values measured on January 1st at 12:00AM from WY 2008 through 2022. By using one streamflow value for each time of day and day of year, the year-to-year variability in streamflow is minimized. Estimates of load were aggregated by month and year to run the Seasonal Kendall test (Webber et al., 2023). It is important to understand that this method was used solely to determine how load changed from 2008 through 2022. The loads determined by this method do not represent the actual load of each constituent exported from the watershed in any given year because each measured value of streamflow replaced with a normalized value.

### Statistical significance of trends

Results from the WRTDS-S method serve as the primary determinant of trends in flow-normalized load in this study. Significance of trends in flow-normalized load using the LOESS-adjustment and streamflow-averaging methods and FN trends in concentration were assessed at a serial correlation adjusted *p*-value of ≤ 0.05 used to denote a very likely trend and 0.05 < × ≤ 0.10 to denote a likely trend (Lorenz, 2017; Yue et al., 2002). Significance of the WRTDS and WRTDS-S trends were assessed with the WRTDS Bootstrap Test (WBT), calculated in R v. 4.3.2 using the EGRETci package (v. 2.0.4, Hirsch et al., 2015a). The WBT, an alternative to null-hypothesis significance testing (NHST), uses a likelihood-based approach to determine the probability that a trend is occurring (Hirsch et al., 2015a). Instead of the traditional *p*-value used for NHST, the WBT computes the posterior mean estimate of the probability ($\widehat{p}$) of an increasing trend. In this study, a trend was describe as very likely decreasing if $\widehat{p}$ ≤ 0.1, likely decreasing if 0.1 < $\widehat{p}$ ≤ 0.33, no trend if 0.33 < $\widehat{p}$ < 0.66, likely increasing if 0.66 ≤ $\widehat{p}$ < 0.90, and very likely increasing if $\widehat{p}$ ≥ 0.90. Hirsch et al. (2015) posit that the likelihood approach is a more rational approach to describing changes in environmental conditions when those conditions are related to determining if a specific management action is needed or if an implementation has achieved an expected outcome.

### Factors affecting nutrient and sediment load

Previously, Webber et al. (2023) evaluated drivers of water-quality and ecological response in 14 of the 20 streams monitored for this study. Linear-mixed effects modeling was used to identify factors that had the greatest effect on spatiotemporal patterns in monitored water-quality responses. A set of predictor variables describing time invariant variables such as physical watershed features (e.g., slope, area), stream geomorphology (e.g., bank height, channel width), soil characteristics (e.g., hydraulic conductivity, soil porosity), and geologic descriptors (e.g., Piedmont, Triassic Lowlands), as well as time-varying predictors such as water quality data (e.g., dissolved oxygen), climate, land cover, roadways, housing units, wastewater infrastructure, and stormwater infrastructure were considered (Webber et al., 2022). This study sought to determine if the factors Webber et al. (2023) identified as the primary drivers of spatiotemporal patterns in SS, TP, and TN concentrations apply to loads at the five intensively monitored stations in Fairfax County (Table S3). Nonparametric Spearman rank correlation testing was used to test for relations between mean annual load and predictor variables. This study acknowledges that the sample size (*n* = 5) limits the statistical power of this analysis. Results are used for the basis of developing a better understanding of these watersheds rather than to claim causal relations.

## Results

### Surrogate model performance

Surrogate-regression models were well calibrated for all three major constituents of interest (SS, TP, and TN; Table S4) explaining over 97% of variability in load and 79 to 99% of variability in concentration for SS and TP. The performance of the TN concentration model varied by station, ranging from 36 to 78%. Concentration and load models generally had high Nash–Sutcliffe efficiency scores (> 0.75), suggesting accurate predictions, and low bias. The partial-load ratio, a measure of underestimation or overestimation, was close to one for all models, indicating low prediction bias.

### Spatial and temporal patterns in load

**Fig. 2:**
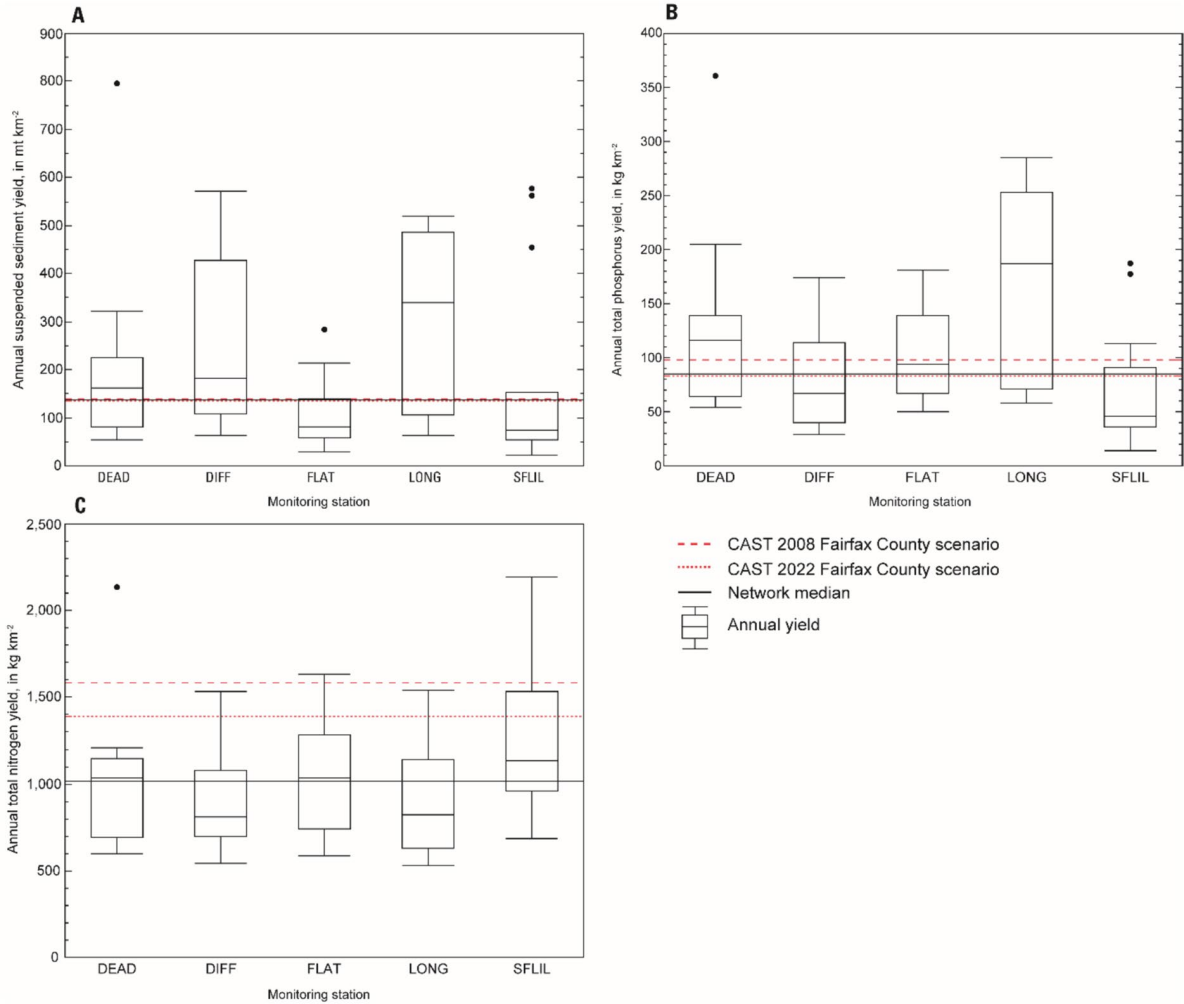
Annual yields of **A** suspended sediment, **B** total phosphorus, and **C** total nitrogen at the five intensive monitoring stations from 2008 through 2022 with comparison to Chesapeake Bay Program’s Chesapeake Assessment Scenario Tool (CAST) scenarios for 2008 and 2022 (Chesapeake Bay Program, 2020)

**Fig. 3:**
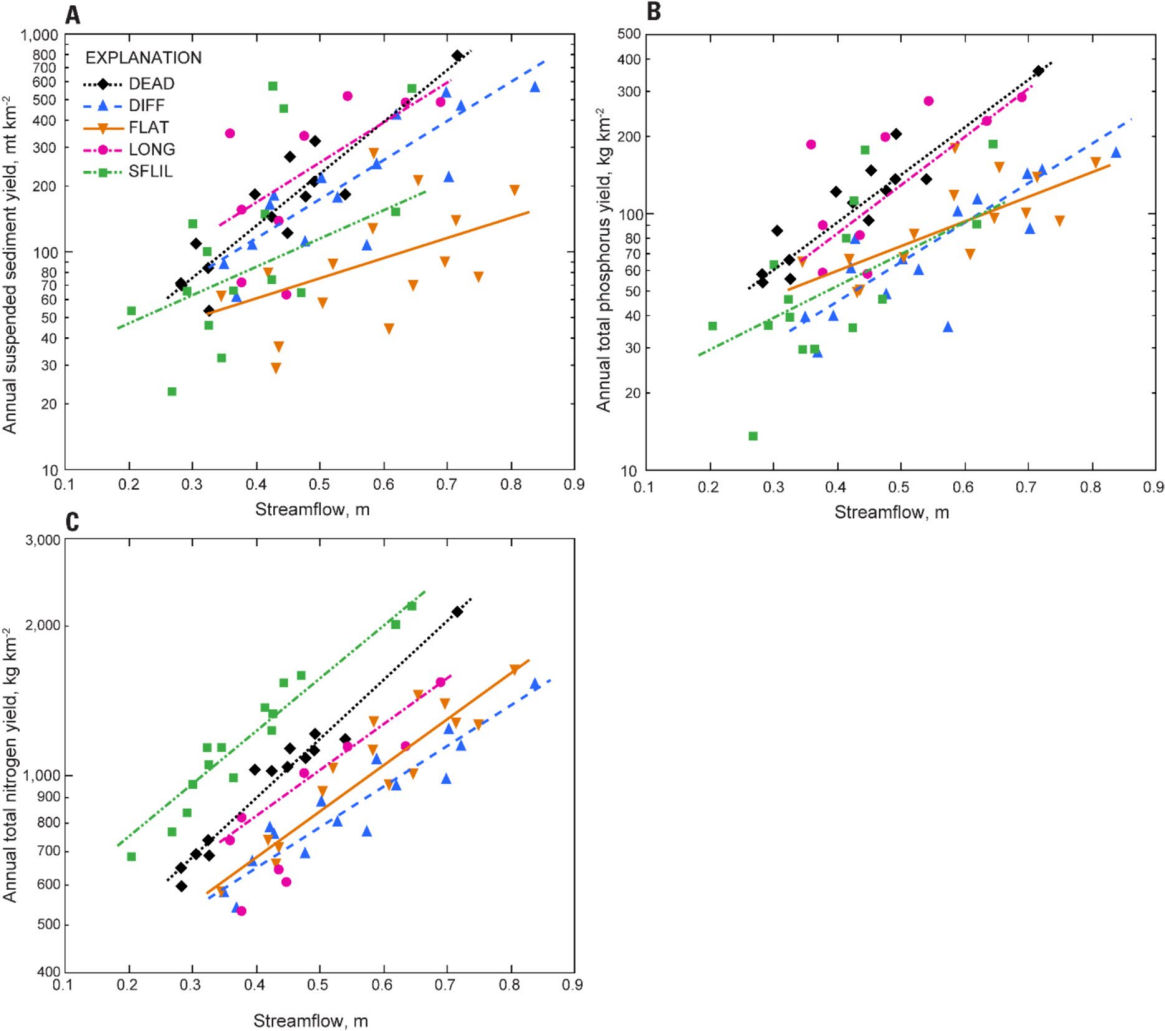
Relation between annual streamflow volume and annual yields of **A** suspended sediments, **B** total phosphorus, and **C** total nitrogen at the five intensively monitored stations

The average annual suspended sediment (SS) load across all stations was 1900 mt, ranging from 160 to 8100 mt (Porter, 2025). Average annual yield, the area-normalized load, typically was highest at LONG and lowest at FLAT. The average network yield, the area-normalized load, is remarkably similar to the county specific rate derived from the 2008 and 2022 CAST model scenarios (Fig. 2A; Chesapeake Bay Program, 2020). The slope of the relation between SS yield and streamflow yield was lower at FLAT than the other four stations (Fig. 3A), meaning that an increase in streamflow relates to a smaller increase in load. The majority (98%) of SS was transported during stormflow events rather than baseflows, and load was highest in spring and summer. Yield was most strongly correlated with predictors describing sediment availability such as bank height, soil depth, and soil texture (clay, sand, and silt content; Table 2). Contrary to Webber et al. (2023), stream density was not strongly correlated with SS yield, and this relation was negative in direction. This contradiction results from a negative correlation between stream density and bank height in these five streams.

**Table 2:** Correlations between sediment and nutrient yields and predictors of spatial variability based on Pearson’s *r* coefficient of determination. *N* = 5 for each comparison. P GEOL is the potential watershed phosphorus contribution from naturally occurring geological materials, and P Soil A is the estimated phosphorus concentration in the Soil A horizon. A full list of predictor variable definitions are provided in Table S3. Bold results are statistically significant at *p*-value ≤ 0.1

Predictor	Suspended sediment	Total particulate phosphorus	Total dissolved phosphorus	Total particulate nitrogen	Total dissolved nitrogen
Bank height	**0.90**	**0.80**	− 0.21	**0.83**	− 0.63
Channel width	− 0.12	0.36	0.76	0.35	− 0.55
Clay	− 0.63	− 0.41	0.59	− 0.36	− 0.02
P GEOL	− 0.66	− 0.15	**0.85**	0.02	0.00
P SOIL A	− 0.19	0.06	0.44	0.26	− 0.08
Soil depth	0.80	0.51	− 0.64	0.45	− 0.14
Sanitary sewer	0.07	0.55	**0.85**	0.72	− 0.73
Sand	0.75	0.44	− 0.63	0.41	− 0.12
Septic density	− 0.09	− 0.50	− 0.84	− 0.68	**0.81**
Silt	** − 0.88**	− 0.44	0.61	− 0.47	0.35
Turfgrass cover	− 0.46	0.19	**0.98**	0.30	− 0.29
Stream density	− 0.56	− 0.45	− 0.21	− 0.68	0.78
Stream length	− 0.50	− 0.57	− 0.43	− 0.79	**0.88**
Daily max WT	− 0.36	0.11	**0.89**	0.20	− 0.46
Daily min DO	0.55	0.13	** − 0.88**	0.01	0.29

The average annual total phosphorus (TP) load across all stations was 950 kg, ranging from 95 to 2800 kg. Annual TP yield typically was highest at DEAD and LONG, but similar at the other three stations. The average network yield is remarkably similar to the county specific rate derived from the 2022 CAST model scenario (Fig. 2B; Chesapeake Bay Program, 2020). The slope of the relation between TP yield and streamflow yield was steepest at DEAD and LONG (Fig. 3B). Like SS, P transport occurred primarily during stormflow events, an average of 91% of TP was exported by stormflows; however, this percentage was slightly lower at FLAT (84%). At all stations, the composition of TP load varied spatially and temporally. Total P load primarily was composed of particulate bound P (average 81%), but at FLAT, orthophosphate was higher, making up 32% of the total. Loads were highest in summer and lowest in fall and winter.

The dissolved and particle fractions of P may originate from different sources and be mobilized and delivered by different mechanisms; therefore, factors affecting spatial variability in load were investigated on these fractions separately. Like SS, particulate P was strongly correlated with bank height and soil depth (Table 2). The dissolved fraction was positively correlated with the percentage of turf grass in the watershed, the potential watershed phosphorus contribution from naturally occurring geologic materials, and the average annual daily maximum water temperature, and negatively correlated with soil depth and the average annual daily minimum dissolved oxygen concentration.

The average annual total nitrogen (TN) load across all stations was 9600 kg, ranging from 3170 to 21,800 kg. Total N yields were similar across these stations. Notably, the average network yield is about 40% lower than the average county specific rate derived from the 2008 and 2022 CAST model scenarios (Fig. 2C; Chesapeake Bay Program, 2020). The slope of the relation between TN yield and streamflow was steepest at SFLIL and DEAD, and more gradual at DIFF and FLAT (Fig. 3C). Transport of TN was more uniformly distributed between stormflows and baseflows than SS and TP with only 57% of the average TN load conveyed during stormflow events; however, this characteristic varied spatially. At LONG, 81% of the load was exported during storms compared to only 37% in SFLIL.

Nitrate accounted for about half (55%) of the TN load at these stations; the remaining half was a mixture of particulate bound N (25%) and dissolved organic N (20%), although this varied spatially and temporally. At SFLIL, nitrate accounted for 78% of the TN load compared to only 32% at LONG, and approximately 50% at the other three stations. Total N loads were more nitrate dominant in winter (70%) than summer (53%). This seasonal shift varied spatially; for example, in winter, nitrate made up 93% of the TN load in SFLIL, but only 47% in LONG. Spatial differences in dissolved N load were correlated with septic system density and stream length, whereas particulate N was correlated with bank height (Table 2).

### Flow-normalized trends in concentration and load

**Fig. 4:**
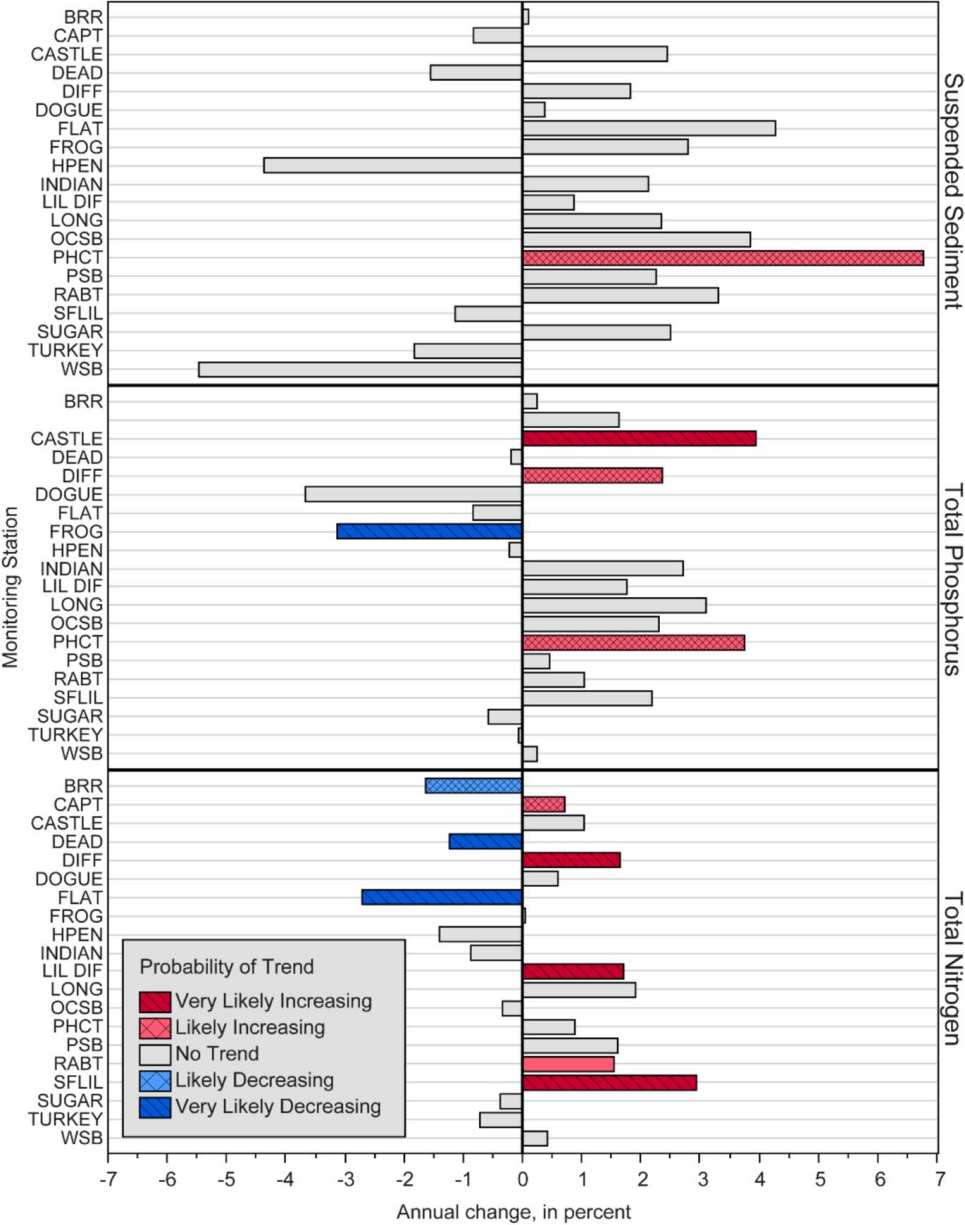
Annual percent change in flow-normalized concentration for suspended sediment, total phosphorus, and total nitrogen at the 20 monitoring stations. Probability of a trend is defined in the methods under *statistical significance of trends*

Concentrations of SS, P, and N were obtained each month at all 20 stations from samples that primarily represent water-quality conditions at baseflow. Concentrations of SS typically were low, ranging from 2 to 5 mg L^−1^, although a wide range of concentrations were observed (0.5–2080 mg L^−1^). Flow-normalized SS concentration increased by nearly 7% per year at PHCT, whereas no change occurred at the other 19 stations (Fig. 4; Table S5).

**Fig. 5:**
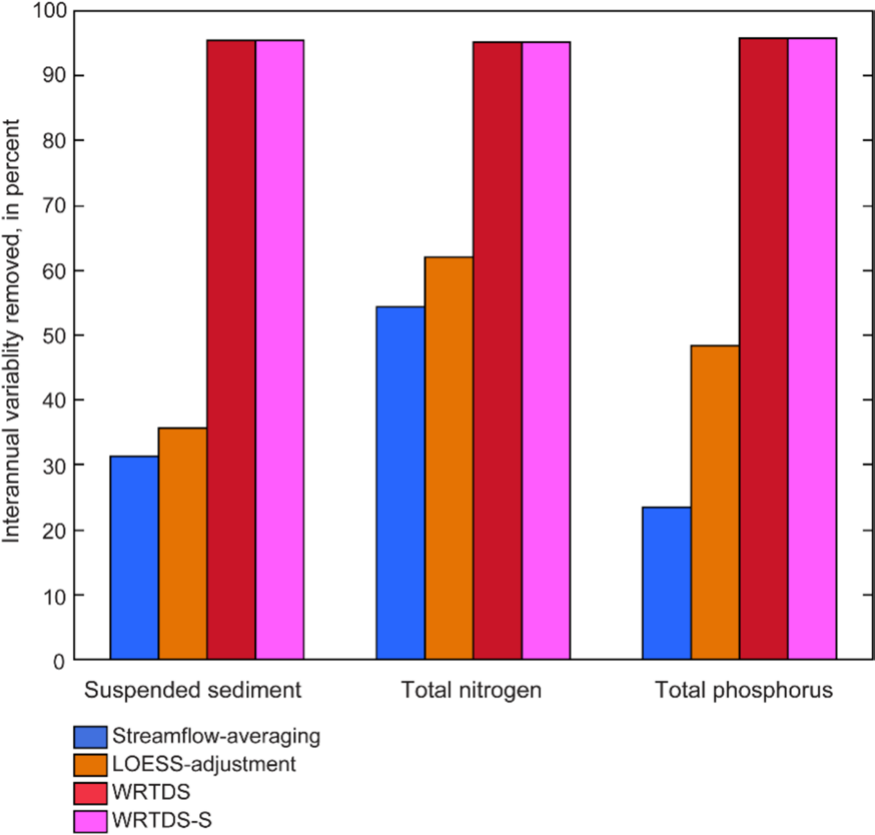
Percentage of interannual variability in annual suspended sediment, total nitrogen, and total phosphorus loads removed by the four flow-normalization and flow-adjustment methods

Nutrient concentrations in monthly samples primarily were in the dissolved form. Concentrations of TP in monthly samples typically ranged from 0.011 to 0.073 mg L^−1^, although concentrations as high as 1.630 mg L^−1^ were observed. Total phosphorus was highest at the three streams draining watersheds located entirely within the Triassic Lowlands physiographic sub-province, FROG, FLAT, and HPEN, although increasing trends were not observed at these stations (Fig. 5). An increase in dissolved P drove the overall increase in TP at PHCT, CASTLE, and DIFF (Table S6, S7). Concentrations of TP were low at all stations, so these percent increases translate to small unit increases of 0.005–0.012 mg L^−1^ over the 15 years of monitoring. The most notable reduction occurred at FROG, where the highest median concentration in the network (0.073 mg L^−1^) decreased by 3.1% per year. Additional insight into changes in TP at each station can be gained by examining trends in the dissolved and particulate fractions provided in the online resources (Table S7, S8, and S9).

Total N concentrations as high as 8.23 mg L^−1^ were observed, but values typically ranged from 1 to 2 mg L^−1^. Spatial variability in median TN concentration was substantial; concentrations of 3 to 5 mg/L were typical at CAPT and SFLIL, whereas values below 2 mg L^−1^ were common at most stations. Total N decreased at three stations, increased at five stations, and typically mirrored patterns in dissolved N, the primary component of N in baseflows (Fig. 5; Tables S10 and S11). Particulate N decreased at most stations; however, concentrations often were lower than the detection limit (Table S12). This degree of censoring reduces the statistical power of these tests and reflects only minor decreases in concentration (0.02–0.04 mg/L over 15 years). The change in dissolved N is confounded by the lack of a trend in either of its components, nitrate and dissolved organic N. Additional insight into changes in TN at each individual station can be gained by examining trends in nitrate, dissolved organic nitrogen, and total organic nitrogen, which are provided as online resources (Tables S13, S14, and S15).

**Fig. 6:**
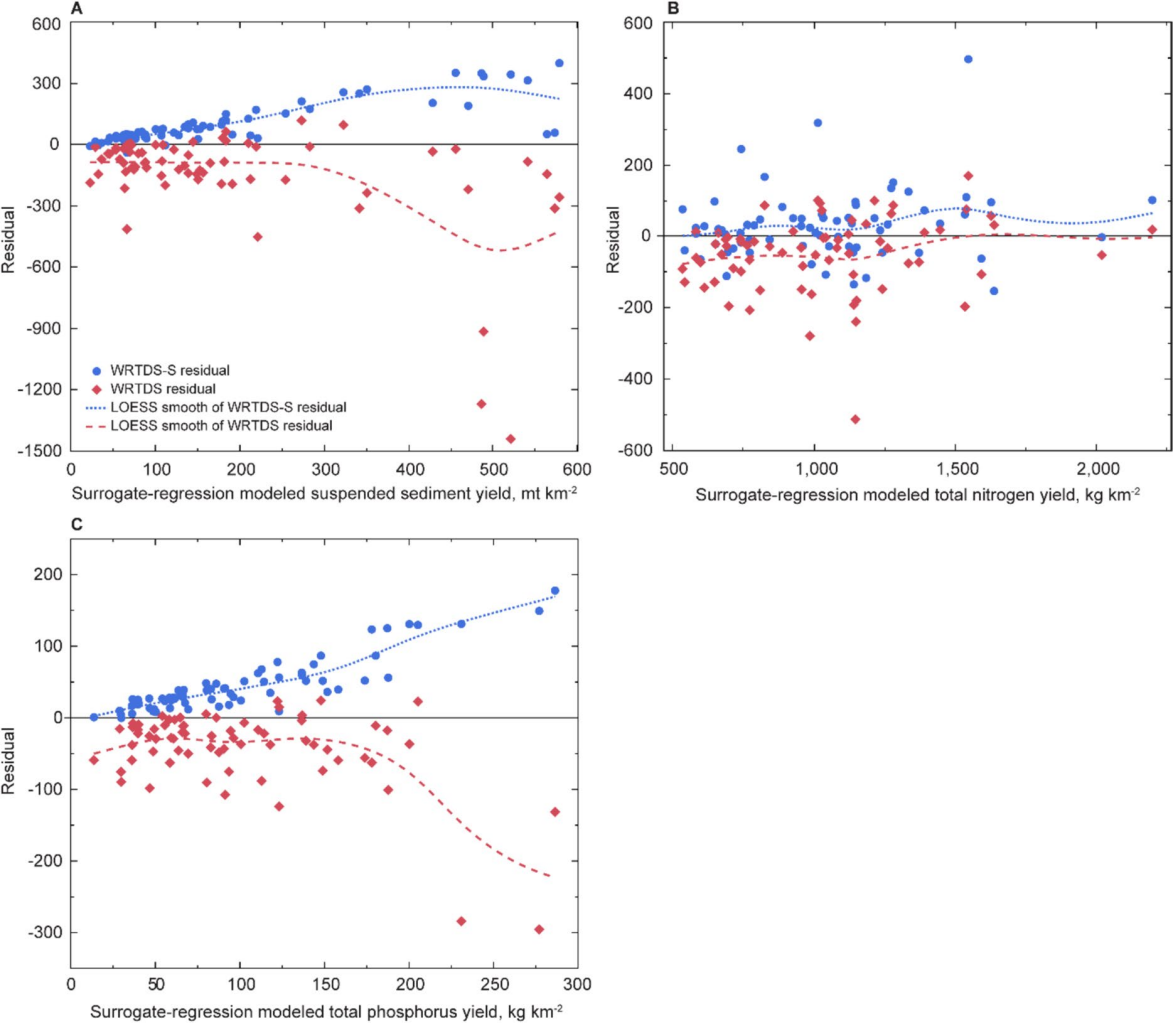
Comparison of surrogate-regression modeled yields of **A** suspended sediment, **B** total nitrogen, and **C** total phosphorus and residuals values. Here, each residual is computed as the difference between the annual surrogate-regression modeled yield and the WRTDS or WRTDS-S non-flow-normalized yield. Loads were converted to yield, area-normalized load, for this analysis because results from all stations were analyzed together

Trends were calculated with four methods designed to reduce streamflow-induced variability in annual load estimates. The two WRTDS methods removed more year-to-year variability than LOESS-adjustment or streamflow-averaging approaches (Fig. 5; S1). Consistency in trend between methods was most common at FLAT and DIFF, the two largest watersheds, and for dissolved constituents across all stations (Tables S16–S26). The two WRTDS methods achieve the same efficiency of flow-normalization given that the process is identical, but WRTDS-S improves load computation by using a mean daily concentration. The relation between surrogate-model-derived loads and WRTDS-S non-flow-normalized loads had a higher *R*^2^ and lower root mean square error than WRTDS for all three primary constituents (SS, TP, and TN; Table 3). Total N loads computed with these two methods were more similar to surrogate-modeled loads, whereas SS and TP consistently were underestimated by WRTDS-S and overestimated by WRTDS (Fig. S1). Divergence in these estimates was greater in years with higher loads (Fig. 6). Although LOESS-adjustment was less efficient at flow-normalization, load estimates are improved through the use of sub-hourly measurements of streamflow and estimates of concentration. Streamflow-averaging is least efficient at flow-normalization and produces a timeseries of FN-load that is only interpretable in the context of trend (i.e., change over time).

**Table 3:** Statistics describing the relation between annual surrogate-model loads and non-flow-normalized WRTDS-S and WRTDS loads. *R*^2^ is the coefficient of determination, RMSE is the root-mean-square error, and MAE is the mean absolute error

Statistic	SS		TN		TP	
	WRTDS-S	WRTDS	WRTDS-S	WRTDS	WRTDS-S	WRTDS
R^2^	0.62	0.60	0.92	0.91	0.70	0.74
RMSE	141	294	103	117	58	70
MAE	101	157	68	83	45	53

**Fig. 7:**
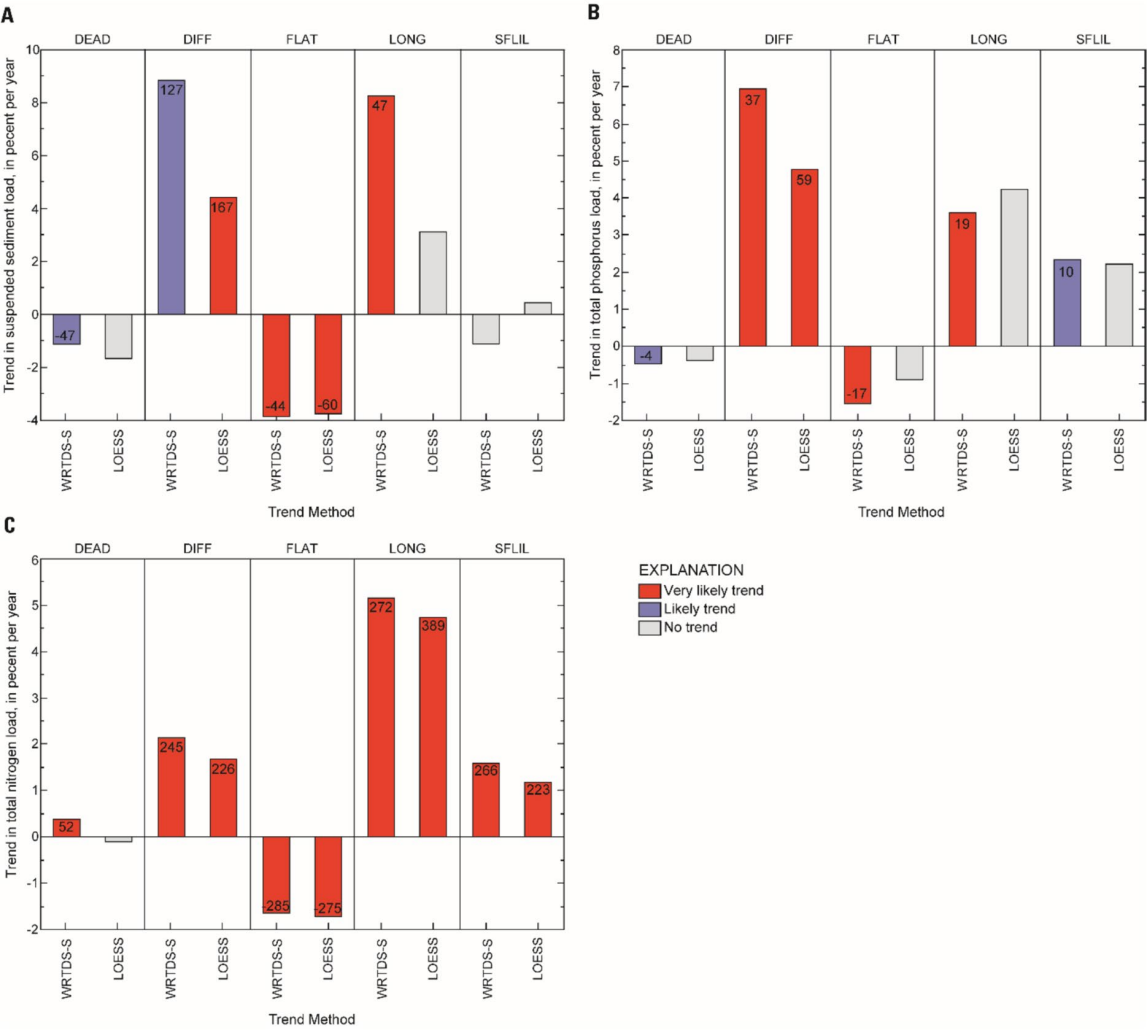
Annual percent change in flow-normalized load for **A** suspended sediment, **B** total phosphorus, and **C** total nitrogen, using two methods to control for streamflow. Likelihood of a trend is defined in methods under *statistical significance of trends*. The total change in yield, area-normalized load, is inset within the bar for instances where a trend was detected

The FN process is identical between WRTDS and WRTDS-S, and the latter was observed to have produced loads more similar to the surrogate-model, so WRTDS-S and LOESS-adjustment are the primary focus of further discussion. Comparison of all four methods is provided as an online resource (Tables S16–S26). The direction and magnitude of trends were similar between these two approaches (Fig. 7). Fewer statistically significant trends were identified using the LOESS-adjustment method, likely due to year-to-year variability that this approach did not fully remove (Figs. 5 and 7; S1).

An annual decrease of nearly 4% in FN-SS load occurred at FLAT, which translates to a total reduction of 480 mt from WY 2008 through WY 2022. A smaller reduction of about 1.1% per year occurred at DEAD (46.6 mt from WY 2009 to 2022). Conversely, FN-SS load increased by about 8.5% per year at DIFF and LONG, a total increase of 1810 mt and 448 mt since WY 2008 and WY2014, respectively.

Flow-normalized TP load decreased at FLAT and DEAD by about 1.5% per year (188 kg since WY 2008) and 0.5% per year (20 kg since WY 2009), respectively. At DIFF, LONG, and SFLIL FN-TP load increased by 2 to 7% per year. Increases at these stations translate to a total unit change of 521 kg at DIFF, 179 kg at LONG, and 68.9 kg at SFLIL.

The FN-TN load increased at four of the five stations and decreased at one. At FLAT, load decreased by about 5% per year or approximately 3000 kg from WY 2008 to WY 2022. Increasing trends at the other four stations occurred at a magnitude of about 0.4 to 5% per year, with the greatest percent change occurring at LONG. The largest unit increases occurred at DIFF (approximately 3500 kg from WY 2008 to WY 2022), and LONG (2600 kg from WY 2014 to WY 2022).

## Discussion

### Patterns in load

Mean annual SS yield in the five intensively monitored watersheds is nearly identical to CAST model scenarios, which indicates the Chesapeake Bay TMDL is properly calibrated for SS in this locality, and likely in others with similar land use and geology. The three most urbanized Piedmont watersheds typically yielded the most SS. Spatial differences were best predicted by bank height, which suggests erosion of bank derived legacy sediment is a key factor affecting SS transport in these watersheds. Legacy sediments are those stored in the landscape due to accelerated erosion from land disturbance following European colonization (Miller et al., 2019). These legacy sediments account for most of the streambank material in many Piedmont streams and represent a significant source of suspended sediment during periods of high streamflow (Gellis et al., 2017; Noe et al., 2020). A previous study determined bank material accounts for 91% of suspended sediments in a Fairfax County stream (Cashman et al., 2018). Equally, yields at FLAT likely are lower due to landscape attributes that differentiate the Triassic Lowlands sub-province from the Piedmont. This region is defined by broad sloping topography, shallow soils, and wide stream channels—factors that limit sediment availability and bank erosion (Noe et al., 2020). These results suggest that historical land use and physiographic features are important factors affecting SS transport. Incorporating historical land use and physiographic features into regulatory models using locally relevant data could increase the effectiveness of managment practices in Fairfax County.

Annual SS loads are well correlated with annual peak streamflow (Porter et al., 2020), with the highest loadings during periods of heavy rainfall. These storms result in rapid fluctuations in the hydrograph, commonly referred to as stream flashiness. Flashiness is positively correlated with impervious cover, making this effect more pronounced in heavily developed watersheds (Porter & Rice, 2024; Porter et al., 2020). The combination of urbanization and sediment availability in the DEAD and LONG watersheds likely contribute to elevated SS loadings. Notably, at SFLIL, a less developed Piedmont watershed (15.8% impervious cover), loads were similar to loads at FLAT in most years, but were among the highest in years with extreme weather events. This suggests that dominant SS sources in SFLIL are floodplain and streambank erosion, or entrainment of coarse particulates (sand) stored in channel deposits (Porter et al., 2020).

A seasonal shift in SS loading, characterized by increased transport in spring and summer months, likely is driven by local weather patterns. In the Mid-Atlantic, convective storm systems are common during the warmest months of the year. These storms can deliver a large volume of rainfall over a short period of time producing stormflows capable of generating large sediment pulses. For example, in June 2015, four heavy rainfall events collectively delivered approximately 143 mm of precipitation to the DEAD watershed, causing the transport of about 67% of the annual SS load. The magnitude and frequency of these events may increase due to changes in climate (Trenberth, 2011; US Global Change Research Program, 2017). Precipitation increases in the mid-Atlantic region are observed by Patterson et al. (2013), Easterling et al. (2017), and Rice et al., (2017, 2023). This change uniquely alters the hydrologic regime of urban watersheds (Porter & Rice, 2024), affecting the full spectrum of storm return intervals (e.g., 2 years, 10 years, 100 years) to a higher magnitude of runoff; however, most management practices are designed to treat smaller, more frequent storms (US EPA, 1999). Practices designed to treat less frequent, higher magnitude storms may be needed to effectively reduce SS loads. This is supported by MacRae (1997), who found management practices designed to treat the 2-year storm had no effect on the rate of erosion of downstream channels.

Mean annual TP yield in the five intensively monitored watersheds is lower than the CAST model scenario in 2008 and similar to the 2022 model scenario, which suggests recent model calibrations have improved the accuracy of P predictions for this area. Phosphorus yields at all five intensive stations consisted mainly of particulate bound P and were positively correlated with streamflow. Similarly, Duan et al. (2012) reported that most TP transport in their study watersheds occurred under high flow conditions and observed a shift in P export to high flows with increasing urbanization. The particulate P yield at FLAT was lower than the other four stations, but was offset by higher dissolved P, primarily orthophosphate. Elevated concentrations and loads of orthophosphate and lower rates of retention in the Triassic Lowlands have been linked to geological inputs and lower phosphorus soil storage capacity due to shallow soil depth (Porter et al., 2020; Webber et al., 2023).

Webber et al. (2023) report that spatial variability in TP concentration in Fairfax County streams is best explained by differences in turf grass cover, soil depth, and dissolved oxygen concentration. The study’s phosphorus data, collected mainly during baseflows, better explain factors affecting dissolved P than the more abundant particulate fraction in TP loads. Strong correlations between particulate P and both bank height and soil depth likely are explained by the well-established relation between sediment and phosphorus transport (Withers & Jarvie, 2008, Houshmand et al., 2014; Fox et al., 2016). This suggests that effectively controlling SS is crucial for managing P loads, which are typically particulate-dominant. Dissolved P loads were correlated with turfgrass, a proxy for the application of P in turf grass fertilizer, which is supported by other studies (Soldat et al., 2009; King et al., 2012; Bachman et al., 2016). High dissolved P loads at FLAT also may be related to P contributions from naturally occurring geologic materials, which are elevated in the Triassic Lowlands (Nardi, 2014; Webber et al., 2023). About 25% of the annual P load at FLAT comes from geologic sources, based on this study’s average load (1082 kg) and Ator’s (2019) estimate of geologic inputs (252 kg). The geology of the Triassic Lowlands is formed of shale and limestone produced from particulate matter of plants and animals and is consequently rich in phosphorus (Froelich & Zenone, 1985b). The geological composition and poorly drained soils, result in anoxic conditions that can lead to iron reduction and the subsequent release of iron-bound phosphorus to the water column (Carey et al., 2022; Lehtoranta & Pitkänen, 2003; Li et al., 2021). Freolich and Zenone (1985b) also propose that the shallow fractured bedrock in this region provides little protection for contaminated surface runoff and may lead to more polluted groundwater.

Total P transport was highest in summer and lowest in winter and fall, patterns consistent across particulate and dissolved fractions. Seasonal variations in hydrology and water temperature explain these patterns. Prior studies have found relations between dissolved P loads and temperature mediated biotic and abiotic processes (Barrow, 1979; Froelich, 1988; James & Barko, 2004; Duan et al., 2012). A strong positive correlation between yield and water temperature and negative correlation to dissolved oxygen may indicate P release from soils at temperatures that produce hypoxia (Yang et al., 2021). Results from this study corroborate those of Duan et al. (2012) who observed higher soluble reactive P concentrations in summer than in winter in a small urban headwater stream outside of Baltimore, MD, USA, located approximately 30 km northeast of the study area. Others have reported elevated concentrations in summer months but low export due to sorption to bed sediments (Bowes et al., 2003). High dissolved P export in summer months in Fairfax County streams may suggest bed sediments are already P saturated. Elevated TP loadings in summer likely are also driven by regional precipitation patterns and the entrainment of SS. The amplified stormflow peaks and the flashy hydrologic regime that results from these high-intensity storms can produce elevated SS transport and P ions adsorbed to those sediments. For this reason, streambank erosion can be a major source of P (Margenot et al., 2023).

Mean annual TN yield in the five intensively monitored watersheds are lower than CAST model predictions suggesting the Chesapeake Bay watershed model currently either overestimates contributions of N from this area or underestimates the effect of management practices. Loads were more strongly correlated with annual streamflow volume than TP and SS, offering insight into differences in source, mobilization, delivery, and ultimately the management of those contaminants. This relation is consistent with other urban stream studies where substantial decreases in N retention were observed between dry and wet years (O’Driscoll et al., 2010; Duncan et al., 2017; Webber et al., 2023). Approximately 75% of TN loads in the monitored Fairfax County streams were dissolved, and unlike SS and TP, transport occurred evenly between baseflows and stormflows. Nitrate, the dominant form of N in these streams, is a highly soluble and mobile anion that does not easily absorb to soil particles and can contaminate groundwater (Jury & Nielsen, 1989). Where groundwater is nitrate rich, surface water-groundwater exchange is an important delivery mechanism, and in wetter years, the mobilization of nitrate from soil porewater and shallow groundwater is enhanced (Webber et al., 2023).

Seasonal differences in TN may be related to nitrate loss through plant uptake and denitrification in warmer months (Duncan et al., 2017; Monti & Scorca, 2002). The degree of seasonal variability in TN varies spatially depending on the dominant form of N in the watershed. For example, at LONG, N is predominantly particulate bound, leading to muted seasonal shifts. Loads are mainly controlled by local weather patterns and the stormflows they generate. In SFLIL, where nitrate from septic effluent is abundant, patterns in loading are more closely related to annual streamflow volume and seasonal shifts in retention (Hyer et al., 2016; Porter et al., 2020). Webber et al. (2023) identified septic density as the main driver of spatial differences in TN concentration in Fairfax County streams and annual rainfall and change in septic density as drivers of temporal variability. As in Webber et al. (2023), dissolved N loads in this study were most strongly correlated with septic density. Like other particulate contaminants, spatial variability in particulate bound N was most strongly correlated with bank height, a proxy for erosion potential and sediment availability, and temporal variability was related to annual streamflow volume. Although N loading is less commonly associated with bank erosion than suspended sediments or P, Noe et al. (2022) report that 6% of the N load from the non-tidal Chesapeake Bay watershed originates from bank erosion and that small headwater streams are net erosional.

### Trends in flow-normalized concentration

Understanding trends in flow-normalized concentration and flow-normalized load are useful in tandem given the different aspects of source, mechanism, and delivery they represent. Loads are most useful for understanding the delivery of contaminants and for evaluating progress towards regulatory benchmarks. Concentrations are better descriptors of source and mechanism and can be used to investigate stressors of aquatic communities such as benthic macroinvertebrates or fish. Trends in load and concentration may not align (Hirsch et al., 2010; Stamm et al., 2014). For example, a decrease in concentration at high streamflows coupled with an increase at low streamflow may lead to a reduction in load, given that higher streamflows transport a disproportionate amount of load. However, this may not enhance the health of aquatic organisms that spend the bulk of their life cycle in baseflow conditions (Herman & Nejadhashemi, 2015).

Trends in FN concentration were evaluated on samples collected each month, typically during baseflows, and therefore largely represent the chemistry of groundwater. Unlike SS, N and P contain dissolved fractions that can be concentrated at baseflow. Trends in FN-TP concentration decreased at FROG, which is consistent with the decline from 2008 to 2018 reported previously (Porter et al., 2020). This pattern is notable because FROG has the highest TP concentrations in the 20-station monitoring network. Decreases in both particulate and dissolved organic P led to this decline. Interestingly, no management practices credited with sediment or nutrient reductions were implemented in this watershed. Webber et al. (2023) report that changes in median annual TP concentration are best explained by annual precipitation, with higher concentrations in dryer years due to conditions that favor mineralization of organic matter, and greater increases in watersheds with deeper soils. If these factors were driving the decreasing trend in FN-TP concentration at FROG, similar results would have been expected at the other stations in the Triassic Lowlands given similar soil depth and climate.

Notable increases in FN-TP and FN-nitrate concentration were observed at CASTLE, the least developed watershed in the network. The trend in TP concentration was driven by an increase in dissolved P despite a decrease in the particulate fraction. Porter et al. (2020) report that this watershed had the highest index of biotic integrity score at the beginning of the monitoring period, indicating “excellent” stream health, but showed a decline through 2018. That study hypothesized this change was related to developmental pressures in the watershed. Over those 10 years, the watershed experienced an increase in low and medium intensity residential development and a 2% increase in impervious cover. As a result, septic density increased by 22% (55 new systems overall) from 44 systems/km^2^ in 2008 to 54 systems/km^2^ in 2022. Ongoing urbanization in this watershed may be contributing to an increase in N and P sources, reducing retention of these nutrients, and exerting stress on sensitive benthic macroinvertebrate communities (Kaye et al., 2006; Reisinger et al., 2016; Porter et al., 2020; Fanelli et al., 2022). The three stations with the largest increase in FN-TN concentration (SFLIL, LIL DIFF, and CAPT) had similarly large increases in FN-nitrate. These three watersheds are among the highest in septic density of those monitored. In both LIL DIFF and CAPT, septic density increased by about 14% from 2008 through 2022, whereas density decreased in SFLIL by 3%.

### Methods to assess trends in flow-normalized load

This study found that WRTDS-S, a method designed as an integration of WRTDS and surrogate-regression modeling, was the most viable option tested for flow-normalizing load in small urban watersheds. The flow-normalization process was identical for both applications of WRTDS, with the only difference being the accuracy of the derived loads. These methods removed substantially more interannual variability than the LOESS-adjustment and streamflow-averaging approaches. The LOESS-adjustment and streamflow-averaging methods offer the benefit of directly utilizing surrogate-modeled derived load and concentration, respectively, but flow-normalizing at a monthly time-step is inadequate for flashy watersheds. The extent of this limitation varies by contaminant. Annual FN loads computed with the LOESS-adjustment and streamflow-averaging methods were less variable for N than SS and P. The streamflow-averaging method yielded annual flow-normalized loads that were substantially lower than from surrogate models, complicating regulatory assessment without the offsetting benefit of effective flow normalization.

WRTDS-S produced a more accurate estimate of annual load than WRTDS due to differences in the temporal resolution of model inputs. Both methods produced TN loads similar to surrogate-regression models; however, SS and TP loads differed substantially, most notably during years when loading was above average. Differences likely result from the use of mean daily streamflow and concentration, which makes these approaches more suited to large watersheds (Zhang & Hirsch, 2019). In small urban watersheds with frequent streamflow fluctuations, daily values may not accurately represent conditions (Robertson et al., 2018). Like the LOESS-adjustment method, this issue is more pronounced for particulate-dominant contaminants (SS and TP) whose peak transport occurs during episodic storm flows (Porter et al., 2020). Total N transport is more strongly correlated to streamflow volume than storm peaks, so a mean daily value is more suitable (Porter et al., 2020; Porter, 2022). WRTDS-S offers improvement over WRTDS for load computation in small watersheds by replacing periodic measures of concentration, which are unlikely to represent the full range of the hydrologic regime, seasons, and time, with daily estimates. However, this method would be improved if it could operate on a sub-daily timestep (e.g., hourly). WRTDS-S provided acceptable results at the intensively monitored watersheds; however, it is important to note that these were the larger watersheds in the study. Whether this method is appropriate for watersheds under 5 km^2^ remains uncertain. Although WRTDS-S predicts the “actual” (i.e., non-flow-normalized) load less accurately than a surrogate-regression model, it remains a functional approach for flow-normalizing load to properly investigate trend. Additional study could investigate the applicability of WRTDS-Kalman Filter (WRTDS-K) in these watersheds. This method is an adaptation of WRTDS that has been shown to improve load predictions by accounting for the autocorrelation structure of model residuals (Zhang & Hirsch, 2019). Unfortunately, WRTDS-K is only designed to compute load, not a flow-normalized load or trend in load. This study therefore concludes that when both load and trend are of interest in a small urban watershed a combination of surrogate-regression methods and WRTDS-S are appropriate.

### Trends in flow-normalized load

Decreasing trends in FN-load occurred at DEAD and FLAT for most constituents. In both watersheds, stream restorations were completed, for which the county received regulatory credits for substantial SS and nutrient reductions. Although this study was not designed to establish a causal relation between management actions and trends in load, these two watersheds had a higher level of management practice implementation than the other three watersheds for which loads were computed. The stream restoration may have contributed to the decline in FN-TN at FLAT; however, this trend occurred steadily over the duration of the study, predating the restoration. To evaluate whether stream restoration drove the decreasing trend in SS and TP at FLAT, a more statistically robust approach that controls for other factors, such as changes in land use, climate, and urban activities, should be applied (Webber et al., 2023). Insight can be gained by comparing trends in FN concentrations to FN loads at this station. Although FN-SS and FN-TP loads decreased, FN-TP concentration did not change, and FN-SS concentration increased. This suggests that load decreases stem from lower concentrations at higher streamflows when the majority of TP and SS are transported. This reduction is likely related to the stream restoration which was designed to lower erosion potential (Fairfax County, 2025).

At DEAD, Fairfax County received credits for the treatment of 115% of the watershed area and 161% of impervious surfaces; however, only small decreases in SS, TP, and nitrate were observed, and TN increased. The slight increase in FN-TN load at DEAD was driven by a rise in dissolved organic N, which was largely offset by declines in nitrate and particulate N. Restorations, which restore hydrologic connectivity with the hyporheic zone and increase hydrologic residence time, have been shown to enhance denitrification (Klocker et al., 2009). The difference in measured response at FLAT and DEAD may be related to the proximity of the monitoring station to the restored reach. The monitoring station is located within the restored reach in FLAT, whereas the downstream extent of restored reach in DEAD is approximately 75 m from the monitoring station. The difference in trends at these two stations suggests that either water-quality improvements are occurring at the reach- rather than watershed-scale, or significant sources of load were not addressed by the stream restoration in DEAD.

An increase in FN-load was observed for most contaminants in watersheds with little to no management practice implementation, although drivers of these trends may differ. At SFLIL, the increase in TN resulted from a similar increase in nitrate. Increasing nitrate in the SFLIL watershed has previously been linked to increasing septic density and annual precipitation (Hyer et al., 2016; Webber et al., 2023). The increase in FN-TN and FN-TP load at LONG and DIFF result from of an increase in both particulate and dissolved N. Particulate N and P trends in these watersheds likely are linked to increasing SS. The increase in FN-SS load at LONG is particularly noteworthy because this watershed is a tributary of Accotink Creek, which is currently regulated under a SS TMDL due to a failure to meet the aquatic life use standard (Interstate Commission on the Potomac River Basin, 2017). To achieve the reductions required under this regulation, Fairfax County has established the Long Branch Central Watershed Management Area Project to restore approximately 75% of the Long Branch stream channel, tributaries, and outfall channels over the coming decade. Beginning in 2021, the USGS partnered with Fairfax County to conduct additional monitoring in this watershed to assess this project.

Factors affecting trends in dissolved nutrients at DIFF and LONG may differ. Like SFLIL, the rise in dissolved N at DIFF may result from nitrate enrichment in groundwater from a growing number of septic systems in the watershed (Hyer et al., 2016; Webber et al., 2023). In contrast, the increase observed at LONG, where septic density is low, may be due to enhanced transport and availability of nitrate resulting from urbanization. The hydrology and geomorphology of LONG reflect classic symptoms of the “urban stream syndrome” (Interstate Commission on the Potomac River Basin, 2017; Porter et al., 2020; Walsh et al., 2005). This condition is characterized by, among others, increased stream flashiness, reduced baseflows, and channel incision (Walsh et al., 2005). These changes can increase the transport of upland nutrient sources, reduce in-stream nutrient removal, and increase organic matter decomposition (Klocker, et al., 2009; Meyer et al., 2005).

## Conclusions

Local governments are investing heavily in management practices to meet local and regional regulatory requirements designed to improve water quality; however, the efficacy of these practices remains poorly understood. This study provides the long-term monitoring data to quantify spatial and temporal patterns in load that are needed to fill this knowledge gap. This study suggests that the current model, CAST, used to assess progress towards the Chesapeake Bay TMDL, is well calibrated for SS and TP in Fairfax County, but may overestimate TN contributions. Nutrient and sediment loads vary across the Fairfax County due to factors related to geology, past land use, and watershed management implementations. Particulate contaminants such as SS, P, and particulate N were highest in streams with steep banks and a history of legacy sediment accumulation. Nitrogen export was greatest in watersheds with a high density of septic systems, whereas P was highest in watersheds located in the Triassic Lowlands sub-province. Reductions in nutrient and sediment loads were observed in watersheds with completed stream restorations, whereas increases occurred for most contaminants in watersheds with few implemented management practices. These results suggest that management actions are having positive effects on water quality, but additional study is needed to determine if actual reductions match credited amounts. This study determined that WRTDS-S, which integrates the surrogate-regression model derived concentrations with robust methods for removing streamflow induced variability, is the most viable available option tested for quantifying trend in flow-normalized load in a small, urbanized watershed.

## Supplementary Information

Below is the link to the electronic supplementary material.

Supplementary file1 (DOCX 564 KB)

## Data Availability

The data that support the findings in this study are openly available from the U.S. Geological Survey National Water Information System database (https://waterdata.usgs.gov/nwis) or can be downloaded from the USGS series Data Release, Porter (2025), accessible at 10.5066/P1NTROI6.

## Declarations

All authors have read, understood, and have complied as applicable with the statement on “Ethical responsibilities of Authors” as found in the Instructions for Authors.
