# Supplementary material for: Spatiotemporal patterns in urban nutrient and suspended sediment loads and stream response to watershed management implementation
Source: Environ Monit Assess. 2025 Apr 1;197(4):497. doi: 10.1007/s10661-025-13917-7 (PMC11961521; doi:10.1007/s10661-025-13917-7)
Supplement: Supplementary file 1 — Supplementary file1 (DOCX 564 KB) [file 10661_2025_13917_MOESM1_ESM.docx]

Supplemental Information

Spatiotemporal patterns in urban nutrient and suspended sediment loads and stream response to watershed management implementation

Environmental Monitoring and Assessment

Authors: Aaron J. Porter^1^*

^1^U.S. Geological Survey, Virginia-West Virginia Water Science Center

1730 East Parham Road, Richmond, VA 23228

*Corresponding author

Corresponding author email: aporter@usgs.gov

Statements and Declarations

This study was funded by Fairfax County, Virginia, and the U.S. Geological Survey. All authors have read, understood, and have complied as applicable with the statement on "Ethical responsibilities of Authors" as found in the Instructions for Authors. The authors declare no known competing financial interests or personal relationships that could have appeared to influence the work reported in this paper. Any use of trade, firm, or product names is for descriptive purposes only and does not imply endorsement by the U.S. Government.

Data sources

All continuous and discrete data were retrieved from the National Water Information System website (NWIS) using the USGS station IDs listed in Table 1 (U.S. Geological Survey, 2023). Monitoring stations have various lengths of record. Records date back to 2008, 2009, or 2013, depending on the station. Years in this study represent water years (WY), defined as October 1 through September 30, and named for the year in which the period ends. Additional datasets not available on NWIS, such as load models and load input files are provided in Porter (2025). Predictor data were obtained from Webber et al. (2022).

Watershed names listed in tables S1 and S3 – S26 and figure S1 are defined in Table 1.

Table S1 Summary of management practices implemented in the study watersheds and the Chesapeake Bay Total Maximum Daily Load credits received from 2009 through 2022 (U.S. Environmental Protection Agency, 2010; Fairfax County 2023; Fairfax County 2024). Only watersheds with implemented practices are listed

| Watershed Name | Treated area, km^2^ | Watershed area treated, % | Impervious treated, km^2^ | Impervious area treated, % | Credited TN reduction, kg yr^-1^ | Credited TP reduction, kg yr^-1^ | Credited TSS reduction, mt yr^-1^ |
| --- | --- | --- | --- | --- | --- | --- | --- |
| FLAT | 10.59 | 97.2 | 3.58 | 103 | 622 | 73 | 37 |
| INDIAN | 10.52 | 166 | 3.99 | 187 | 184 | 41 | 13 |
| DEAD | 6.10 | 115 | 2.62 | 161 | 1150 | 312 | 111 |
| DIFF | 1.80 | 12.7 | 0.70 | 16.5 | 643 | 121 | 56 |
| OCSB | 1.49 | 39. | 1.05 | 56.7 | 168 | 100 | 30 |
| BRR | 0.54 | 6.18 | 0.40 | 10.1 | 160 | 25 | 27 |
| LONG | 0.44 | 4.59 | 0.15 | 6.45 | 143 | 52 | 18 |
| TURKEY | 0.44 | 6.58 | 0.11 | 5.44 | 213 | 58 | 20 |
| PSB | 0.44 | 8.90 | 0.12 | 8.70 | 450 | 190 | 23 |
| SUGAR | 0.08 | 4.99 | 0.01 | 4.53 | 64 | 31 | 11 |
| HPEN | 0.05 | 1.49 | 0.03 | 3.40 | 11 | 1 | 2 |
| WSB | 0.03 | 1.20 | 0.01 | 0.86 | 12 | 1 | 1 |
| DOGUE | 0.01 | 0.590 | 0.01 | 2.00 | 10 | 1 | 1 |

Table S2 List of constituents analyzed from water quality samples

| Constituent | Method |
| --- | --- |
| Suspended sediment concentration | ASTM D3977-97^a^ |
| Total phosphorus | SM425C & 425E^b^ |
| Total dissolved phosphorus | SM425C & 425E^b^ |
| Total particulate phosphorus | SM425C & 425E^b^ |
| Orthophosphate | USEPA 365.2^c^ |
| Total nitrogen | USEPA 351.2 + USEPA 353.2^d,e^ |
| Total dissolved nitrogen | USEPA 351.2 + USEPA 353.2^d,e^ |
| Total particulate nitrogen | USEPA 351.2 + USEPA 353.2^d,e^ |
| Total Kjeldahl nitrogen | USEPA 351.2^f^ |
| Dissolved total Kjeldahl nitrogen | USEPA 351.2^f^ |
| Nitrate + nitrite | USEPA 351.2^f^ |

^a^ASTM International, 2019

^b^American Public Health Association (APHA), American Water Works Association (AWWA), & Water Environment Federation (WEF), 2011

^c^U.S. Environmental Protection Agency (EPA), 1974

^d^U.S. Environmental Protection Agency (EPA), 1978a

^e^U.S. Environmental Protection Agency (EPA), 1978b

^f^U.S. Environmental Protection Agency (EPA), 1978c

Table S3 Description of predictor variables used to hypothesize drivers of spatial patterns in yield in Fairfax County, Virginia. Data were obtained from Webber et al., 2022

| Predictor name | Predictor definition |
| --- | --- |
| Bank height | Average streambank height, in meters |
| Channel width | Average stream channel width, in meters |
| Septic density | Density of all septic systems, in number of units per square kilometer |
| Sanitary sewer | Density of sanitary-sewer lines, in kilometers per square kilometer |
| P SOIL A | Estimated phosphorus concentration in the Soil A horizon, in milligrams per kilogram |
| P GEOL | Potential watershed phosphorus contribution from naturally occurring geological materials in milligrams per kilogram |
| Soil depth | High value for the range of total soil thickness, in cm |
| Sand content | Area- and depth-weighted sand content of soil, in percent |
| Clay | Area- and depth-weighted clay content of soil, in percent |
| Silt content | Area- and depth-weighted silt content of soil, in percent |
| Turfgrass cover | Watershed area classified as turfgrass, in percent |
| Stream density | Length of perennial streams, in kilometers per square kilometer |
| Stream length | Length of perennial streams, in kilometers |
| Daily max WT | Mean annual daily maximum water temperature, in degrees Celsius |
| Daily min DO | Mean annual daily minimum dissolved oxygen concentration, in milligrams per liter |

Table S4 Surrogate regression model diagnostics. R^2^ is the adjusted coefficient of determination, NSE is the Nash-Sutcliffe Efficiency score as defined by (Nash and Sutcliffe, 1970), which provides a measure of model fit that ranges from 1 (perfect) to negative infinity. Bias is the percentage by which the model overestimates (positive) or underestimates (negative) the sum of the estimated loads compared with the sum of the observed loads; PLR is the partial load ratio is a ratio of the sum of the estimated loads to the sum of the observed loads, which indicates load overestimation (>1) or underestimation (<1). Residual variance is the variance in the observed and predicted values, and N is the number of samples used to calibrate the model.

| Parameter | Station | Load Model | | | | Concentration Model | | | | Residual  Variance | N |
| --- | --- | --- | --- | --- | --- | --- | --- | --- | --- | --- | --- |
|  |  | R^2^ | NSE | Bias, % | PLR | R^2^ | NSE | Bias, % | PLR |  |  |
| Suspended  Sediment | DEAD | 97.7 | 0.46 | 6.0 | 1.30 | 92.6 | 0.35 | 4.4 | 1.01 | 0.43 | 535 |
|  | DIFF | 97.4 | 0.27 | 0.8 | 1.00 | 93.0 | 0.06 | -11.0 | 0.88 | 0.43 | 498 |
|  | FLAT | 97.9 | 0.24 | 3.0 | 1.30 | 99.0 | 0.65 | 11.0 | 1.10 | 0.33 | 541 |
|  | LONG | 97.8 | 0.78 | -2.4 | 0.98 | 92.9 | 0.57 | -0.4 | 0.99 | 0.47 | 312 |
|  | SFLIL | 97.8 | 0.44 | 8.6 | 1.10 | 94.2 | 0.72 | 0.3 | 1.00 | 0.36 | 504 |
| Total  Phosphorus | DEAD | 98.2 | 0.83 | 2.5 | 1.00 | 88.4 | 0.65 | 1.3 | 1.01 | 0.21 | 465 |
|  | DIFF | 97.1 | 0.81 | 0.6 | 0.99 | 87.8 | 0.62 | -0.8 | 0.99 | 0.26 | 441 |
|  | FLAT | 97.2 | 0.74 | 2.3 | 0.99 | 79.4 | 0.54 | -0.5 | 1.00 | 0.22 | 466 |
|  | LONG | 97.6 | 0.95 | -0.4 | 0.99 | 87.1 | 0.74 | 1.3 | 1.01 | 0.30 | 269 |
|  | SFLIL | 96.8 | 0.79 | 5.3 | 1.10 | 89.1 | 0.71 | 2.3 | 1.02 | 0.33 | 459 |
| Total Nitrogen | DEAD | 98.5 | 0.89 | -1.3 | 0.99 | 36.0 | 0.32 | -0.3 | 0.99 | 0.08 | 466 |
|  | DIFF | 98.0 | 0.88 | -4.4 | 0.96 | 53.0 | 0.51 | -0.2 | 1.01 | 0.08 | 454 |
|  | FLAT | 98.2 | 0.89 | 1.5 | 1.02 | 47.5 | 0.41 | 0.0 | 1.00 | 0.09 | 460 |
|  | LONG | 98.8 | 0.95 | -5.2 | 0.95 | 78.4 | 0.7 | 0.1 | 1.00 | 0.09 | 271 |
|  | SFLIL | 98.1 | 0.86 | -0.8 | 0.99 | 38.6 | 0.39 | -0.3 | 1.00 | 0.06 | 427 |

Table S5 Trends in suspended sediment concentration. Statistical significance is assessed at p-value ≤ 0.1. Statistically significant trends are in bold, with increases in orange and decreases in green

| Station | Number of observations | Percent  censored  observations | Median Concentration | Non-flow-normalized | | | Flow-normalized | | |
| --- | --- | --- | --- | --- | --- | --- | --- | --- | --- |
|  |  |  |  | Total change^a^ | Annual percent change | p-value | Total change^a^ | Annual percent change | p-value |
| BRR | 167 | 2.4 | 4.0 | 0.000 | 0.00 | 0.5454 | 0.061 | 0.10 | 0.9650 |
| CAPT | 165 | 8.1 | 3.0 | 0.000 | 0.00 | 0.2722 | -0.371 | -0.82 | 0.7406 |
| CASTLE | 166 | 3.5 | 4.0 | 0.000 | 0.00 | 0.4792 | 1.470 | 2.45 | 0.4940 |
| DEAD | 169 | 5.2 | 3.0 | 0.000 | 0.00 | 0.9104 | -0.697 | -1.55 | 0.2441 |
| DIFF | 166 | 2.3 | 4.0 | 0.000 | 0.00 | 0.3755 | 1.096 | 1.83 | 0.2038 |
| DOGUE | 117 | 4.2 | 4.0 | 0.000 | 0.00 | 0.8296 | 0.152 | 0.38 | 0.8685 |
| FLAT | 169 | 6.9 | 3.0 | 0.682 | 1.52 | 0.2365 | 1.924 | 4.28 | 0.1236 |
| FROG | 167 | 8.7 | 2.0 | 0.000 | 0.00 | 0.1725 | 0.840 | 2.80 | 0.1752 |
| HPEN | 114 | 7.8 | 3.0 | 0.000 | 0.00 | 0.2951 | -1.441 | -4.37 | 0.2697 |
| INDIAN | 162 | 1.7 | 4.0 | **1.590** | **2.65** | **0.0308** | 1.278 | 2.13 | 0.1927 |
| LIL DIFF | 164 | 0.6 | 4.0 | 0.000 | 0.00 | 0.5522 | 0.525 | 0.87 | 0.5936 |
| LONG | 117 | 4.2 | 4.0 | 0.000 | 0.00 | 0.8972 | 0.940 | 2.35 | 0.4143 |
| OCSB | 161 | 7.7 | 3.0 | 0.000 | 0.00 | 0.2236 | 1.734 | 3.85 | 0.1418 |
| PHCT | 166 | 4.7 | 5.0 | **3.765** | **5.02** | **0.0925** | **5.084** | **6.78** | **0.0793** |
| PSB | 151 | 0.6 | 5.0 | 0.000 | 0.00 | 1.0000 | 1.695 | 2.26 | 0.2182 |
| RABT | 97 | 4.1 | 4.0 | 0.000 | 0.00 | 0.6995 | 1.324 | 3.31 | 0.5808 |
| SFLIL | 165 | 2.3 | 4.0 | 0.000 | 0.00 | 0.6763 | -0.681 | -1.14 | 0.6258 |
| SUGAR | 117 | 10.8 | 3.0 | 0.000 | 0.00 | 0.7869 | 0.751 | 2.50 | 0.5615 |
| TURKEY | 139 | 8.9 | 2.0 | 0.000 | 0.00 | 0.5914 | -0.548 | -1.83 | 0.3226 |
| WSB | 118 | 4.2 | 3.0 | 0.000 | 0.00 | 0.1808 | -1.639 | -5.46 | 0.1309 |

^a^ total change is based on the number of years the station was in operation, as described in table 1, ranging from 10-15 year

Table S6 Trends in total phosphorus concentration. Statistical significance is assessed at p-value ≤ 0.1. Statistically significant trends are in bold, with increases in orange and decreases in green

| Station | Number of observations | Percent  censored  observations | Median Concentration | Non-flow-normalized | | | Flow-normalized | | |
| --- | --- | --- | --- | --- | --- | --- | --- | --- | --- |
|  |  |  |  | Total change^a^ | Annual percent change | p-value | Total change^a^ | Annual percent change | p-value |
| BRR | 169 | 0 | 0.028 | 0.000 | 0.00 | 0.8274 | 0.001 | 0.25 | 0.7425 |
| CAPT | 172 | 0 | 0.015 | **0.007** | **3.23** | **0.0512** | 0.004 | 1.63 | 0.3710 |
| CASTLE | 173 | 0 | **0.020** | **0.010** | **3.39** | **0.0222** | **0.012** | **3.95** | **0.0080** |
| DEAD | 173 | 0 | 0.030 | 0.003 | 0.57 | 0.6882 | -0.001 | -0.19 | 0.8196 |
| DIFF | 174 | 0 | **0.014** | **0.006** | **2.83** | **0.0160** | **0.005** | **2.37** | **0.0719** |
| DOGUE | 120 | 0 | 0.031 | -0.016 | -5.01 | 0.1713 | -0.011 | -3.66 | 0.2153 |
| FLAT | 173 | 0 | 0.060 | -0.015 | -1.63 | 0.2331 | -0.008 | -0.84 | 0.6126 |
| FROG | 173 | 0 | 0.073 | **-0.035** | **-3.17** | **0.0054** | **-0.034** | **-3.13** | **0.0127** |
| HPEN | 114 | 0 | 0.052 | 0.002 | 0.36 | 0.6818 | -0.001 | -0.22 | 0.9437 |
| INDIAN | 171 | 0 | 0.016 | 0.006 | 2.37 | 0.3831 | 0.007 | 2.72 | 0.2995 |
| LIL DIFF | 170 | 0 | 0.015 | 0.005 | 2.25 | 0.1695 | 0.004 | 1.76 | 0.2518 |
| LONG | 120 | 0 | 0.022 | 0.000 | 0.00 | 0.8988 | 0.007 | 3.10 | 0.3168 |
| OCSB | 169 | 0 | 0.019 | 0.004 | 1.46 | 0.4265 | 0.007 | 2.31 | 0.1291 |
| PHCT | 169 | 0 | 0.018 | **0.010** | **3.70** | **0.0369** | **0.010** | **3.76** | **0.0632** |
| PSB | 161 | 0 | 0.031 | 0.000 | 0.00 | 1.0000 | 0.002 | 0.46 | 0.8596 |
| RABT | 97 | 0 | 0.033 | -0.010 | -3.03 | 0.3052 | 0.003 | 1.05 | 0.8037 |
| SFLIL | 174 | 0 | 0.013 | 0.003 | 1.61 | 0.1421 | 0.004 | 2.19 | 0.1010 |
| SUGAR | 119 | 0 | 0.017 | 0.000 | 0.00 | 0.9023 | -0.001 | -0.58 | 0.7780 |
| TURKEY | 146 | 0 | 0.011 | 0.003 | 1.61 | 0.1731 | -0.000 | -0.06 | 0.9556 |
| WSB | 119 | 0 | 0.017 | 0.009 | 5.15 | 0.0570 | 0.000 | 0.25 | 1.0000 |

^a^ total change is based on the number of years the station was in operation, as described in table 1, ranging from 10-15 years

Table S7 Trends in total dissolved phosphorus concentration. Statistical significance is assessed at p-value ≤ 0.1. Statistically significant trends are in bold, with increases in orange and decreases in green. Bold red text indicates datasets with greater than 50% censored values

| Station | Number of observations | Percent  censored  observations | Median Concentration | Non-flow-normalized | | | Flow-normalized | | |
| --- | --- | --- | --- | --- | --- | --- | --- | --- | --- |
|  |  |  |  | Total change^a^ | Annual percent change | p-value | Total change^a^ | Annual percent change | p-value |
| BRR | 169 | 39.0 | 0.013 | 0.000 | 0.00 | 0.4660 | **0.005** | **2.47** | **0.0501** |
| CAPT | 172 | 47.0 | 0.008 | **0.005** | **4.17** | **0.0196** | **0.006** | **5.35** | **0.0102** |
| CASTLE | 173 | 38.0 | 0.010 | **0.008** | **5.00** | **0.0127** | **0.011** | **7.07** | **0.0043** |
| DEAD | 173 | 31.0 | 0.020 | 0.006 | 1.88 | 0.1943 | 0.004 | 1.22 | 0.3552 |
| DIFF | 174 | **59.0** | 0.007 | 0.000 | 0.00 | 0.2250 | **0.007** | **6.38** | **0.0080** |
| DOGUE | 120 | 29.0 | 0.018 | **-0.016** | **-8.89** | **0.0661** | -0.008 | -4.23 | 0.1315 |
| FLAT | 173 | 30.0 | 0.040 | **-0.024** | **-3.96** | **0.0231** | **-0.021** | **-3.56** | **0.0443** |
| FROG | 173 | 22.0 | 0.059 | **-0.028** | **-3.20** | **0.0090** | **-0.029** | **-3.29** | **0.0197** |
| HPEN | 114 | 8.0 | 0.042 | **0.000** | **0.00** | **0.9762** | 0.005 | 1.17 | 0.6412 |
| INDIAN | 171 | **50.0** | 0.008 | 0.004 | 3.13 | 0.1682 | **0.006** | **4.93** | **0.0591** |
| LIL DIFF | 170 | **50.0** | 0.007 | 0.003 | 3.30 | 0.0309 | **0.007** | **6.93** | **0.0099** |
| LONG | 120 | 33.0 | 0.014 | 0.000 | 0.00 | 0.8464 | 0.005 | 3.55 | 0.3683 |
| OCSB | 169 | 46.0 | 0.011 | 0.001 | 0.91 | 0.4418 | **0.004** | **2.52** | **0.0177** |
| PHCT | 169 | 44.0 | 0.008 | 0.005 | 4.17 | 0.0059 | **0.007** | **6.25** | **0.0119** |
| PSB | 161 | 41.0 | 0.013 | 0.002 | 0.96 | 0.6102 | 0.006 | 3.05 | 0.2275 |
| RABT | 97 | 16.0 | 0.020 | -0.005 | -2.50 | 0.5651 | -0.002 | -0.76 | 0.6148 |
| SFLIL | 174 | **51.0** | 0.007 | **0.004** | **4.08** | **0.0094** | **0.008** | **7.79** | **0.0011** |
| SUGAR | 119 | 73.0 | 0.004 | -0.003 | -6.25 | 0.3679 | **0.004** | **10.55** | **0.1013** |
| TURKEY | 146 | 40.0 | 0.007 | **0.005** | **4.76** | **0.0189** | **0.004** | **4.06** | **0.0522** |
| WSB | 119 | 47.0 | 0.010 | -0.001 | -1.21 | 0.8628 | 0.001 | 0.76 | 0.8054 |

^a^ total change is based on the number of years the station was in operation, as described in table 1, ranging from 10-15 years

Table S8 Trends in total particulate phosphorus concentration. Statistical significance is assessed at p-value ≤ 0.1. Statistically significant trends are in bold, with increases in orange and decreases in green. Bold red text indicates datasets with greater than 50% censored values

| Station | Number of observations | Percent  censored  observations | Median Concentration | Non-flow-normalized | | | Flow-normalized | | |
| --- | --- | --- | --- | --- | --- | --- | --- | --- | --- |
|  |  |  |  | Total change^a^ | Annual percent change | p-value | Total change^a^ | Annual percent change | p-value |
| BRR | 135 | 20.6 | 0.011 | **-0.011** | **-6.82** | **0.0069** | -0.003 | -2.05 | 0.4830 |
| CAPT | 148 | 27.3 | 0.007 | -0.002 | -2.04 | 0.2514 | **0.004** | **3.93** | **0.0453** |
| CASTLE | 149 | 28.3 | 0.008 | -0.004 | -3.13 | 0.1768 | 0.005 | 4.31 | 0.1340 |
| DEAD | 148 | 27.6 | 0.008 | **-0.005** | **-4.17** | **0.0444** | 0.001 | 0.91 | 0.7124 |
| DIFF | 144 | 24.7 | 0.008 | **-0.008** | **-6.25** | **0.0375** | **0.005** | **3.99** | **0.0964** |
| DOGUE | 120 | 35.0 | 0.011 | 0.003 | 2.60 | 0.3620 | **0.006** | **5.29** | **0.0374** |
| FLAT | 132 | 17.8 | 0.010 | 0.000 | 0.00 | 0.7308 | **0.013** | **9.31** | **0.0597** |
| FROG | 144 | 24.9 | 0.008 | **-0.006** | **-4.69** | **0.0796** | 0.002 | 1.73 | 0.6352 |
| HPEN | 114 | 38.3 | 0.010 | 0.004 | 3.75 | 0.2774 | -0.008 | -7.63 | 0.1457 |
| INDIAN | 146 | 26.7 | 0.008 | -0.004 | -3.13 | 0.4376 | 0.004 | 3.30 | 0.1891 |
| LIL DIFF | 151 | 30.0 | 0.007 | **-0.008** | **-7.14** | **0.0971** | 0.001 | 1.42 | 0.7531 |
| LONG | 120 | 35.0 | 0.010 | 0.000 | 0.00 | 0.9467 | 0.010 | 10.26 | 0.1632 |
| OCSB | 142 | 26.0 | 0.007 | -0.002 | -1.59 | 0.5033 | 0.005 | 4.34 | 0.1889 |
| PHCT | 143 | 27.2 | 0.007 | -0.005 | -4.76 | 0.3048 | 0.005 | 5.06 | 0.1987 |
| PSB | 129 | 20.5 | 0.013 | -0.010 | -5.13 | 0.1129 | 0.008 | 3.93 | 0.2486 |
| RABT | 97 | 29.6 | 0.014 | -0.006 | -3.97 | 0.4431 | 0.006 | 4.60 | 0.2316 |
| SFLIL | 151 | 28.7 | 0.007 | **-0.005** | **-4.76** | **0.0613** | 0.002 | 1.90 | 0.3354 |
| SUGAR | 118 | 38.3 | 0.012 | -0.005 | -4.17 | 0.1048 | -0.004 | -3.20 | 0.3476 |
| TURKEY | 138 | 28.8 | 0.007 | 0.000 | 0.00 | 0.6798 | **0.004** | **3.50** | **0.0823** |
| WSB | 119 | **58.0** | 0.005 | 0.004 | 8.00 | 0.6800 | -0.006 | -11.39 | 0.2741 |

^a^ total change is based on the number of years the station was in operation, as described in table 1, ranging from 10-15 years

Table S9 Trends in orthophosphate concentration. Statistical significance is assessed at p-value ≤ 0.1. Statistically significant trends are in bold, with increases in orange and decreases in green

| Station | Number of observations | Percent  censored  observations | Median Concentration | Non-flow-normalized | | | Flow-normalized | | |
| --- | --- | --- | --- | --- | --- | --- | --- | --- | --- |
|  |  |  |  | Total change^a^ | Annual percent change | p-value | Total change^a^ | Annual percent change | p-value |
| BRR | 170 | 45.9 | 0.009 | 0.000 | 0.00 | 0.9649 | -0.001 | -0.46 | 0.7883 |
| CAPT | 172 | 46.5 | 0.010 | 0.002 | 1.54 | 0.4901 | 0.000 | 0.09 | 0.9039 |
| CASTLE | 173 | 41.0 | 0.011 | 0.003 | 2.02 | 0.2469 | 0.004 | 2.15 | 0.1806 |
| DEAD | 174 | 37.9 | 0.018 | 0.008 | 2.78 | 0.4181 | 0.001 | 0.20 | 0.9415 |
| DIFF | 174 | **54.0** | 0.007 | -0.004 | -3.57 | 0.3979 | 0.000 | -0.28 | 0.9332 |
| DOGUE | 120 | 32.5 | 0.014 | **-0.013** | **-8.93** | **0.0930** | -0.010 | -7.03 | 0.1303 |
| FLAT | 173 | 35.1 | 0.032 | -0.012 | -2.50 | 0.2279 | **-0.022** | **-4.57** | **0.0279** |
| FROG | 173 | 33.5 | 0.053 | **-0.017** | **-2.10** | **0.0978** | **-0.041** | **-5.19** | **0.0030** |
| HPEN | 114 | 7.0 | 0.046 | **-0.009** | **-1.89** | **0.0806** | 0.000 | 0.00 | 1.0000 |
| INDIAN | 172 | 49.4 | 0.009 | -0.001 | -0.79 | 0.9303 | 0.001 | 0.47 | 0.7583 |
| LIL DIFF | 170 | **51.2** | 0.008 | -0.002 | -1.39 | 0.7886 | 0.001 | 0.72 | 0.5710 |
| LONG | 120 | 37.5 | 0.012 | -0.006 | -4.76 | 0.1755 | 0.000 | 0.19 | 0.9802 |
| OCSB | 169 | 45.0 | 0.010 | 0.001 | 0.91 | 0.6086 | 0.000 | 0.26 | 0.8193 |
| PHCT | 169 | 46.8 | 0.010 | 0.000 | 0.00 | 0.6317 | 0.000 | 0.02 | 0.9683 |
| PSB | 161 | 44.7 | 0.010 | 0.003 | 2.00 | 0.3611 | 0.004 | 2.55 | 0.1036 |
| RABT | 97 | 12.2 | 0.019 | -0.010 | -5.26 | 0.2265 | -0.006 | -3.18 | 0.4141 |
| SFLIL | 174 | **51.2** | 0.008 | 0.000 | 0.00 | 0.8758 | 0.001 | 0.54 | 0.6608 |
| SUGAR | 120 | **68.3** | 0.005 | -0.007 | -13.81 | 0.2853 | -0.001 | -2.56 | 0.5285 |
| TURKEY | 146 | **50.0** | 0.007 | 0.000 | 0.00 | 0.9865 | -0.001 | -1.17 | 0.5137 |
| WSB | 119 | 39.5 | 0.011 | **-0.010** | **-9.09** | **0.0633** | 0.002 | 2.21 | 0.2951 |

^a^ total change is based on the number of years the station was in operation, as described in table 1, ranging from 10-15 years

Table S10 Trends in total nitrogen concentration. Statistical significance is assessed at p-value ≤ 0.1. Statistically significant trends are in bold, with increases in orange and decreases in green

| Station | Number of observations | Percent  censored  observations | Median Concentration | Non-flow-normalized | | | Flow-normalized | | |
| --- | --- | --- | --- | --- | --- | --- | --- | --- | --- |
|  |  |  |  | Total change^a^ | Annual percent change | p-value | Total change^a^ | Annual percent change | p-value |
| BRR | 169 | 0 | 0.84 | -0.17 | -1.35 | 0.1372 | **-0.21** | **-1.63** | **0.0689** |
| CAPT | 172 | 0 | 4.90 | 0.29 | 0.39 | 0.2816 | **0.53** | **0.72** | **0.0502** |
| CASTLE | 173 | 0 | 1.50 | **0.27** | **1.22** | **0.0690** | 0.24 | 1.05 | 0.1411 |
| DEAD | 174 | 0 | 2.40 | **-0.43** | **-1.20** | **0.0309** | **-0.44** | **-1.23** | **0.0160** |
| DIFF | 174 | 0 | 1.60 | **0.53** | **2.19** | **0.0405** | **0.40** | **1.65** | **0.0483** |
| DOGUE | 120 | 0 | 2.60 | 0.23 | 0.88 | 0.1361 | 0.16 | 0.60 | 0.3616 |
| FLAT | 173 | 0 | 1.47 | **-0.58** | **-2.61** | **0.0072** | **-0.60** | **-2.71** | **0.0095** |
| FROG | 173 | 0 | 1.80 | 0.01 | 0.03 | 0.8377 | 0.01 | 0.05 | 0.9570 |
| HPEN | 114 | 0 | 1.83 | -0.21 | -1.14 | 0.3551 | -0.26 | -1.40 | 0.2290 |
| INDIAN | 172 | 0 | 0.99 | -0.08 | -0.55 | 0.3643 | -0.13 | -0.87 | 0.1639 |
| LIL DIFF | 170 | 0.6 | 2.40 | **0.62** | **1.73** | **0.0215** | **0.62** | **1.71** | **0.0273** |
| LONG | 120 | 0 | 1.04 | 0.26 | 2.45 | 0.2236 | 0.20 | 1.91 | 0.1522 |
| OCSB | 169 | 0 | 1.63 | -0.07 | -0.30 | 0.3908 | -0.08 | -0.34 | 0.3618 |
| PHCT | 169 | 0 | 2.30 | **0.70** | **2.02** | **0.0065** | 0.31 | 0.89 | 0.1054 |
| PSB | 161 | 0 | 1.00 | 0.00 | 0.00 | 0.9636 | 0.24 | 1.61 | 0.1928 |
| RABT | 97 | 0 | 1.20 | 0.16 | 1.35 | 0.2639 | **0.19** | **1.55** | **0.0921** |
| SFLIL | 173 | 0 | 3.08 | **1.38** | **2.98** | **0.0014** | **1.36** | **2.94** | **0.0008** |
| SUGAR | 118 | 0 | 1.62 | 0.04 | 0.27 | 0.7860 | -0.06 | -0.37 | 0.7994 |
| TURKEY | 146 | 0 | 1.00 | -0.02 | -0.11 | 0.7620 | -0.11 | -0.72 | 0.1962 |
| WSB | 119 | 0 | 1.48 | **0.27** | **1.81** | **0.0634** | 0.06 | 0.42 | 0.4336 |

^a^ total change is based on the number of years the station was in operation, as described in table 1, ranging from 10-15 years

Table S11 Trends in total dissolved nitrogen concentration. Statistical significance is assessed at p-value ≤ 0.1. Statistically significant trends are in bold, with increases in orange and decreases in green

| Station | Number of observations | Percent  censored  observations | Median Concentration | Non-flow-normalized | | | Flow-normalized | | |
| --- | --- | --- | --- | --- | --- | --- | --- | --- | --- |
|  |  |  |  | Total change^a^ | Annual percent change | p-value | Total change^a^ | Annual percent change | p-value |
| BRR | 170 | 0 | 0.77 | -0.06 | -0.54 | 0.4124 | -0.06 | -0.51 | 0.4225 |
| CAPT | 172 | 0 | 4.89 | 0.45 | 0.61 | 0.1420 | **0.62** | **0.85** | **0.0311** |
| CASTLE | 173 | 0 | 1.40 | **0.40** | **1.93** | **0.0068** | **0.35** | **1.67** | **0.0092** |
| DEAD | 174 | 0 | 2.30 | -0.32 | -0.91 | 0.1240 | **-0.35** | **-1.02** | **0.0575** |
| DIFF | 174 | 0 | 1.50 | **0.61** | **2.71** | **0.0120** | **0.50** | **2.20** | **0.0122** |
| DOGUE | 120 | 0 | 2.53 | 0.28 | 1.11 | 0.1606 | 0.08 | 0.33 | 0.5847 |
| FLAT | 173 | 0 | 1.37 | **-0.38** | **-1.84** | **0.0671** | **-0.40** | **-1.96** | **0.0230** |
| FROG | 173 | 0 | 1.70 | 0.13 | 0.52 | 0.5445 | 0.19 | 0.74 | 0.5066 |
| HPEN | 114 | 0 | 1.76 | -0.17 | -0.98 | 0.5181 | -0.15 | -0.84 | 0.5651 |
| INDIAN | 172 | 0 | 0.89 | 0.04 | 0.32 | 0.5672 | 0.00 | -0.02 | 1.0000 |
| LIL DIFF | 170 | 0.6 | 2.30 | **0.83** | **2.41** | **0.0093** | **0.91** | **2.65** | **0.0079** |
| LONG | 119 | 0 | 0.96 | 0.25 | 2.64 | 0.2740 | **0.24** | **2.48** | **0.0440** |
| OCSB | 169 | 0 | 1.60 | 0.01 | 0.04 | 0.5882 | 0.05 | 0.22 | 0.4063 |
| PHCT | 169 | 0 | 2.24 | **0.72** | **2.13** | **0.0049** | 0.35 | 1.05 | 0.1141 |
| PSB | 161 | 0 | 0.92 | 0.13 | 0.92 | 0.2963 | **0.32** | **2.33** | **0.0304** |
| RABT | 97 | 0 | 1.13 | 0.16 | 1.41 | 0.2875 | 0.15 | 1.30 | 0.2686 |
| SFLIL | 174 | 0 | 3.00 | **1.45** | **3.21** | **0.0009** | **1.42** | **3.16** | **0.0005** |
| SUGAR | 119 | 0 | 1.52 | 0.12 | 0.80 | 0.2402 | 0.07 | 0.47 | 0.6069 |
| TURKEY | 146 | 0 | 0.95 | 0.07 | 0.50 | 0.4424 | 0.05 | 0.36 | 0.7564 |
| WSB | 119 | 0 | 1.45 | **0.39** | **2.67** | **0.0487** | **0.16** | **1.09** | **0.0727** |

^a^ total change is based on the number of years the station was in operation, as described in table 1, ranging from 10-15 years

Table S12 Trends in total particulate nitrogen concentration. Statistical significance is assessed at p-value ≤ 0.1. Statistically significant trends are in bold, with increases in orange and decreases in green. Bold red text indicates datasets with greater than 50% censored values

| Station | Number of observations | Percent  censored  observations | Median Concentration | Non-flow-normalized | | | Flow-normalized | | |
| --- | --- | --- | --- | --- | --- | --- | --- | --- | --- |
|  |  |  |  | Total change^a^ | Annual percent change | p-value | Total change^a^ | Annual percent change | p-value |
| BRR | 169 | **54.1** | 0.031 | **-0.053** | **-11.39** | **0.0005** | **-0.048** | **-10.40** | **0.0008** |
| CAPT | 172 | **55.2** | 0.049 | **-0.026** | **-3.60** | **0.0004** | **-0.032** | **-4.37** | **0.0001** |
| CASTLE | 173 | **59.0** | 0.048 | **-0.023** | **-3.15** | **0.0004** | **-0.022** | **-3.07** | **0.0023** |
| DEAD | 174 | **55.8** | 0.050 | **-0.018** | **-2.46** | **0.0019** | **-0.024** | **-3.25** | **0.0016** |
| DIFF | 174 | **56.3** | 0.047 | **-0.030** | **-4.26** | **0.0011** | **-0.027** | **-3.74** | **0.0027** |
| DOGUE | 120 | **64.2** | 0.012 | -0.006 | -5.07 | 0.2348 | -0.005 | -4.49 | 0.1516 |
| FLAT | 173 | **56.3** | 0.046 | **-0.041** | **-6.02** | **0.0007** | **-0.038** | **-5.59** | **0.0005** |
| FROG | 173 | **56.7** | 0.047 | **-0.026** | **-3.69** | **0.0014** | **-0.027** | **-3.84** | **0.0006** |
| HPEN | 114 | **58.3** | 0.027 | **-0.043** | **-16.05** | **0.0135** | **-0.043** | **-16.00** | **0.0086** |
| INDIAN | 172 | **59.3** | 0.046 | **-0.041** | **-5.87** | **0.0002** | **-0.041** | **-5.87** | **0.0003** |
| LIL DIFF | 170 | **57.1** | 0.046 | **-0.037** | **-5.45** | **0.0015** | **-0.037** | **-5.36** | **0.0008** |
| LONG | 119 | **78.3** | 0.010 | -0.004 | -4.00 | 0.1742 | -0.001 | -1.07 | 0.7283 |
| OCSB | 169 | **58.0** | 0.046 | **-0.031** | **-4.55** | **0.0006** | **-0.031** | **-4.50** | **0.0007** |
| PHCT | 169 | **58.0** | 0.046 | **-0.020** | **-2.91** | **0.0031** | **-0.020** | **-2.85** | **0.0237** |
| PSB | 161 | **59.6** | 0.046 | **-0.030** | **-4.36** | **0.0003** | **-0.023** | **-3.28** | **0.0110** |
| RABT | 97 | **69.4** | 0.030 | 0.000 | 0.00 | 0.8893 | 0.002 | 0.75 | 0.2866 |
| SFLIL | 173 | **57.5** | 0.050 | **-0.020** | **-2.70** | **0.0014** | **-0.022** | **-2.88** | **0.0013** |
| SUGAR | 118 | **55.8** | 0.031 | **-0.028** | **-8.82** | **0.0377** | **-0.042** | **-13.35** | **0.0564** |
| TURKEY | 146 | **57.5** | 0.049 | **-0.025** | **-3.37** | **0.0003** | **-0.023** | **-3.14** | **<.0001** |
| WSB | 119 | **77.3** | 0.009 | **-0.005** | **-5.84** | **0.0312** | **-0.005** | **-5.40** | **0.0767** |

^a^ total change is based on the number of years the station was in operation, as described in table 1, ranging from 10-15 years

Table S13 Trends in nitrate plus nitrite concentration. Statistical significance is assessed at p-value ≤ 0.1. Statistically significant trends are in bold, with increases in orange and decreases in green

| Station | Number of observations | Percent  censored  observations | Median Concentration | Non-flow-normalized | | | Flow-normalized | | |
| --- | --- | --- | --- | --- | --- | --- | --- | --- | --- |
|  |  |  |  | Total change^a^ | Annual percent change | p-value | Total change^a^ | Annual percent change | p-value |
| BRR | 169 | 1.2 | 0.45 | -0.11 | -1.59 | 0.1730 | -0.10 | -1.47 | 0.1612 |
| CAPT | 172 | 0 | 4.84 | **0.52** | **0.71** | **0.0530** | **0.86** | **1.19** | **0.0105** |
| CASTLE | 173 | 0 | 1.19 | **0.30** | **1.69** | **0.0506** | **0.25** | **1.38** | **0.0968** |
| DEAD | 174 | 0 | 2.03 | -0.38 | -1.25 | 0.1157 | -0.25 | -0.82 | 0.1988 |
| DIFF | 174 | 0 | 1.17 | **0.43** | **2.46** | **0.0507** | **0.33** | **1.90** | **0.0621** |
| DOGUE | 119 | 0 | 2.26 | **0.49** | **2.15** | **0.0571** | 0.48 | 2.14 | 0.1208 |
| FLAT | 173 | 0.6 | 0.93 | **-0.48** | **-3.47** | **0.0145** | **-0.50** | **-3.62** | **0.0091** |
| FROG | 173 | 0 | 1.33 | -0.01 | -0.03 | 0.9840 | 0.01 | 0.03 | 0.9919 |
| HPEN | 114 | 1.7 | 1.30 | -0.26 | -1.98 | 0.3293 | -0.05 | -0.40 | 0.8404 |
| INDIAN | 172 | 0.6 | 0.65 | -0.09 | -0.88 | 0.4102 | -0.08 | -0.87 | 0.2825 |
| LIL DIFF | 169 | 0.6 | 2.01 | **0.84** | **2.77** | **0.0155** | **0.89** | **2.95** | **0.0162** |
| LONG | 120 | 0.8 | 0.59 | 0.27 | 4.65 | 0.1290 | **0.17** | **2.95** | **0.0910** |
| OCSB | 169 | 0 | 1.31 | -0.11 | -0.55 | 0.6206 | -0.09 | -0.47 | 0.4609 |
| PHCT | 168 | 0 | 2.00 | **0.62** | **2.07** | **0.0345** | 0.19 | 0.62 | 0.4974 |
| PSB | 160 | 3.1 | 0.55 | 0.14 | 1.66 | 0.2339 | **0.29** | **3.54** | **0.0151** |
| RABT | 97 | 0 | 0.75 | 0.20 | 2.66 | 0.1500 | 0.18 | 2.38 | 0.2768 |
| SFLIL | 174 | 0 | 2.86 | **1.62** | **3.78** | **0.0006** | **1.54** | **3.59** | **0.0008** |
| SUGAR | 118 | 0 | 1.01 | 0.29 | 2.83 | 0.1238 | 0.22 | 2.18 | 0.1113 |
| TURKEY | 145 | 0 | 0.73 | -0.03 | -0.28 | 0.8072 | -0.03 | -0.26 | 0.7273 |
| WSB | 119 | 0 | 1.12 | **0.40** | **3.60** | **0.0366** | **0.25** | **2.23** | **0.0482** |

^a^ total change is based on the number of years the station was in operation, as described in table 1, ranging from 10-15 years

Table S14 Trends in total Kjeldahl nitrogen concentration. Statistical significance is assessed at p-value ≤ 0.1. Statistically significant trends are in bold, with increases in orange and decreases in green

| Station | Number of observations | Percent  censored  observations | Median Concentration | Non-flow-normalized | | | Flow-normalized | | |
| --- | --- | --- | --- | --- | --- | --- | --- | --- | --- |
|  |  |  |  | Total change^a^ | Annual percent change | p-value | Total change^a^ | Annual percent change | p-value |
| BRR | 168 | 0.6 | 0.36 | -0.07 | -1.38 | 0.2690 | -0.06 | -1.06 | 0.1652 |
| CAPT | 172 | 20.9 | 0.09 | -0.13 | -9.35 | <.0001 | **-0.16** | **-11.63** | **0.0002** |
| CASTLE | 173 | 4.6 | 0.24 | 0.00 | 0.00 | 0.9726 | 0.01 | 0.22 | 0.8873 |
| DEAD | 174 | 8.6 | 0.36 | -0.16 | -2.91 | 0.1166 | **-0.17** | **-3.22** | **0.0181** |
| DIFF | 174 | 1.2 | 0.35 | 0.01 | 0.10 | 0.8171 | 0.02 | 0.32 | 0.7548 |
| DOGUE | 119 | 21.7 | 0.37 | -0.07 | -1.87 | 0.2209 | -0.05 | -1.46 | 0.5142 |
| FLAT | 173 | 1.2 | 0.43 | -0.03 | -0.41 | 0.6845 | 0.00 | -0.04 | 0.9669 |
| FROG | 173 | 1.7 | 0.37 | 0.07 | 1.34 | 0.3156 | 0.05 | 0.82 | 0.3977 |
| HPEN | 114 | 0 | 0.46 | -0.02 | -0.50 | 0.5785 | **-0.13** | **-2.86** | **0.0391** |
| INDIAN | 172 | 1.7 | 0.29 | -0.05 | -1.07 | 0.3726 | -0.05 | -1.07 | 0.3157 |
| LIL DIFF | 169 | 16.5 | 0.24 | **-0.30** | **-8.54** | **0.0038** | **-0.27** | **-7.68** | **0.0015** |
| LONG | 120 | 0.8 | 0.29 | -0.03 | -0.88 | 0.6291 | 0.02 | 0.70 | 0.8654 |
| OCSB | 169 | 3.6 | 0.29 | -0.06 | -1.41 | 0.3288 | -0.03 | -0.58 | 0.6293 |
| PHCT | 168 | 10.1 | 0.27 | -0.07 | -1.80 | 0.2002 | -0.02 | -0.54 | 0.7476 |
| PSB | 160 | 1.2 | 0.37 | -0.05 | -0.82 | 0.4850 | 0.00 | -0.07 | 0.8991 |
| RABT | 97 | 1.0 | 0.32 | -0.08 | -2.54 | 0.1537 | 0.02 | 0.55 | 0.8323 |
| SFLIL | 173 | 20.7 | 0.18 | **-0.29** | **-10.90** | **0.0006** | **-0.28** | **-10.25** | **0.0008** |
| SUGAR | 119 | 0 | 0.54 | **-0.17** | **-3.23** | **0.0425** | **-0.18** | **-3.33** | **0.0378** |
| TURKEY | 145 | 1.4 | 0.25 | -0.07 | -1.75 | 0.2160 | -0.06 | -1.55 | 0.1084 |
| WSB | 119 | 1.7 | 0.34 | -0.06 | -1.77 | 0.1544 | **-0.16** | **-4.74** | **0.0114** |

^a^ total change is based on the number of years the station was in operation, as described in table 1, ranging from 10-15 years

Table S15 Trends in dissolved total Kjeldahl nitrogen concentration. Statistical significance is assessed at p-value ≤ 0.1. Statistically significant trends are in bold, with increases in orange and decreases in green

| Station | Number of observations | Percent  censored  observations | Median Concentration | Non-flow-normalized | | | Flow-normalized | | |
| --- | --- | --- | --- | --- | --- | --- | --- | --- | --- |
|  |  |  |  | Total change^a^ | Annual percent change | p-value | Total change^a^ | Annual percent change | p-value |
| BRR | 169 | 1.8 | 0.30 | 0.05 | 1.18 | 0.2380 | **0.05** | **1.20** | **0.0292** |
| CAPT | 172 | 22.7 | 0.08 | **-0.10** | **-7.84** | **0.0005** | **-0.11** | **-9.14** | **0.0004** |
| CASTLE | 173 | 5.2 | 0.22 | **0.15** | **4.41** | **0.0165** | **0.14** | **4.21** | **0.0144** |
| DEAD | 174 | 10.3 | 0.32 | 0.00 | -0.09 | 0.9329 | -0.05 | -0.96 | 0.5498 |
| DIFF | 174 | 2.9 | 0.31 | **0.11** | **2.45** | **0.0198** | **0.10** | **2.26** | **0.0093** |
| DOGUE | 119 | 28.3 | 0.33 | -0.09 | -2.75 | 0.1163 | -0.08 | -2.46 | 0.2634 |
| FLAT | 173 | 2.3 | 0.38 | **0.11** | **1.88** | **0.0515** | **0.11** | **1.98** | **0.0164** |
| FROG | 173 | 2.9 | 0.33 | **0.15** | **3.03** | **0.0519** | 0.12 | 2.48 | 0.1144 |
| HPEN | 113 | 0 | 0.42 | 0.00 | 0.00 | 1.0000 | **-0.08** | **-1.93** | **0.0335** |
| INDIAN | 172 | 2.3 | 0.26 | **0.12** | **2.98** | **0.0318** | **0.11** | **2.72** | **0.0297** |
| LIL DIFF | 169 | 17.7 | 0.18 | **-0.09** | **-3.35** | **0.0739** | **-0.12** | **-4.31** | **0.0446** |
| LONG | 119 | 0.8 | 0.28 | -0.04 | -1.30 | 0.5591 | 0.01 | 0.18 | 0.8666 |
| OCSB | 169 | 4.7 | 0.27 | 0.09 | 2.16 | 0.2958 | **0.14** | **3.35** | **0.0322** |
| PHCT | 168 | 13.6 | 0.22 | 0.03 | 1.01 | 0.6297 | 0.08 | 2.56 | 0.1396 |
| PSB | 160 | 2.5 | 0.33 | 0.06 | 1.29 | 0.2440 | 0.08 | 1.63 | 0.1799 |
| RABT | 97 | 3.1 | 0.31 | -0.10 | -3.23 | 0.1511 | -0.01 | -0.35 | 0.9337 |
| SFLIL | 174 | 23.6 | 0.11 | **-0.16** | **-9.39** | **0.0005** | **-0.15** | **-9.29** | **0.0004** |
| SUGAR | 119 | 0 | 0.48 | -0.09 | -1.83 | 0.1076 | -0.13 | -2.60 | 0.1040 |
| TURKEY | 145 | 2.7 | 0.23 | 0.07 | 2.06 | 0.1658 | 0.06 | 1.66 | 0.2326 |
| WSB | 119 | 4.2 | 0.30 | -0.06 | -2.04 | 0.1537 | **-0.10** | **-3.25** | **0.0468** |

^a^ total change is based on the number of years the station was in operation, as described in table 1, ranging from 10-15 years

Table S16 Trends in flow normalized suspended sediment loads computed with four different methods. Likelihood of a trend for weighted regressions on time, discharge, and season (WRTDS)_and WRTDS-S is based on the WRTDS bootstrapping test (Hirsch et al., 2015**)** whereas likelihood for Locally Estimated Scatterplot Smoothing (LOESS)--adjustment and streamflow-averaging is based on null-hypothesis significance test where 0.05 < p-value ≤ 0.10 is a likely trend and p-value ≤ 0.05 is a very likely trend

| Method | Measure | DEAD | DIFF | FLAT | LONG | SFLIL |
| --- | --- | --- | --- | --- | --- | --- |
| WRTDS-S | Total Change in load, mt | 46.6 | 1,810 | -479 | 448 | -117 |
|  | Total Change in yield, mt/km^2^ | -8.79 | 127 | -43.9 | 46.7 | -16.7 |
|  | Annual Change, % | -1.12 | 8.85 | -3.86 | 8.26 | -1.11 |
|  | Likelihood of Trend | Likely | Likely | Very Likely | Very likely | No Trend |
| WRTDS | Total Change in load, mt | -191 | 2070 | -1490 | 0 | -1790 |
|  | Total Change in yield, mt/km^2^ | -36 | 146 | -137 | 0 | -256 |
|  | Annual Change, % | -1.48 | 2.85 | -4.37 | -0.22 | -3.73 |
|  | Likelihood of Trend | Likely | Likely | Very Likely | No Trend | Very Likely |
| LOESS  Adjustment | Total Change in load, mt | -247 | 2330 | -649 | 784 | 80 |
|  | Total Change in yield, mt/km^2^ | -46.6 | 164 | -59.5 | 81.7 | 11.4 |
|  | Annual Change, % | -1.66 | 4.43 | -3.75 | 3.12 | 0.14 |
|  | Likelihood of Trend | No Trend | Very Likely | Very Likely | No Trend | No Trend |
| Streamflow  Averaging | Total Change in load, mt | 1.05 | 2.48 | -1.33 | -1.1 | -3.83 |
|  | Total Change in yield, mt/km^2^ | 0.19 | 0.17 | -0.12 | -0.11 | -0.55 |
|  | Annual Change, % | 0.79 | 2.43 | -3.27 | -4.08 | -1 |
|  | Likelihood of Trend | No Trend | Likely | Very Likely | No Trend | No Trend |

Table S17 Trends in flow normalized total phosphorus loads computed with four different methods. Likelihood of a trend for WRTDS-S and WRTDS is based on the WRTDS bootstrapping test (Hirsch et al., 2015**)** whereas likelihood for LOESS-adjustment and Streamflow-averaging is based on null-hypothesis significance test where 0.05 < p-value ≤ 0.10 is a likely trend and p-value ≤ 0.05 is a very likely trend

| Method | Measure | DEAD | DIFF | FLAT | LONG | SFLIL |
| --- | --- | --- | --- | --- | --- | --- |
| WRTDS-S | Total Change in load, kg | -20.2 | 521 | -188 | 179 | 69.8 |
|  | Total Change in yield, kg/km^2^ | -3.82 | 36.7 | -17.3 | 18.7 | 9.97 |
|  | Annual Change, % | -0.479 | 6.95 | -1.55 | 3.60 | 2.34 |
|  | Likelihood of Trend | Likely | Very Likely | Very Likely | Very Likely | Likely |
| WRTDS | Total Change in load, kg | 39.9 | 494 | -250 | 680 | -85.7 |
|  | Total Change in yield, kg/km^2^ | 7.54 | 34.8 | -22.9 | 70.9 | -12.2 |
|  | Annual Change, % | 0.464 | 2.57 | -1.19 | 1.59 | -0.600 |
|  | Likelihood of Trend | No Trend | Likely | Likely | Very Likely | No Trend |
| LOESS  Adjustment | Total Change in load, kg | -2.97 | 69.8 | -12.2 | 49.9 | 13.3 |
|  | Total Change in yield, kg/km^2^ | -0.561 | 4.91 | -1.12 | 5.20 | 1.90 |
|  | Annual Change, % | -0.383 | 4.78 | -0.901 | 4.23 | 2.22 |
|  | Likelihood of Trend | No Trend | Very Likely | No Trend | No Trend | No Trend |
| Streamflow  Averaging | Total Change in load, kg | 0.254 | 2.38 | -0.931 | -0.00603 | 0.703 |
|  | Total Change in yield, kg/km^2^ | 0.0480 | 0.168 | -0.0854 | -0.000628 | 0.100 |
|  | Annual Change, % | 0.579 | 2.09 | -0.455 | -0.0121 | 1.34 |
|  | Likelihood of Trend | No Trend | Very Likely | Very Likely | No Trend | Very Likely |

Table S18 Trends in flow normalized total particulate phosphorus loads computed with four different methods. Likelihood of a trend for WRTDS-S and WRTDS is based on the WRTDS bootstrapping test (Hirsch et al., 2015**)** whereas likelihood for LOESS-adjustment and Streamflow-averaging is based on null-hypothesis significance test where 0.05 < p-value ≤ 0.10 is a likely trend and p-value ≤ 0.05 is a very likely trend

| Method | Measure | DEAD | DIFF | FLAT | LONG | SFLIL |
| --- | --- | --- | --- | --- | --- | --- |
| WRTDS-S | Total Change in load, kg | -13.9 | 574 | -9.73 | 196 | 68.5 |
|  | Total Change in yield, kg/km^2^ | -2.63 | 40.4 | -0.893 | 20.4 | 9.79 |
|  | Annual Change, % | -0.479 | 9.35 | -0.133 | 5.53 | 2.27 |
|  | Likelihood of Trend | Likely | Very Likely | No Trend | Likely | Likely |
| WRTDS | Total Change in load, kg | 65.4 | 488 | -192 | 59.3 | -49.5 |
|  | Total Change in yield, kg/km^2^ | 12.3 | 34.3 | -17.6 | 6.18 | -7.07 |
|  | Annual Change, % | 0.879 | 2.83 | -1.12 | 3.28 | -0.427 |
|  | Likelihood of Trend | No Trend | Likely | Likely | Likely | No Trend |
| LOESS  Adjustment | Total Change in load, kg | -1.89 | 74.0 | 16.5 | 40.3 | 13.5 |
|  | Total Change in yield, kg/km^2^ | -0.357 | 5.21 | 1.52 | 4.20 | 1.94 |
|  | Annual Change, % | -0.299 | 5.58 | 1.73 | 3.69 | 2.20 |
|  | Likelihood of Trend | No Trend | Very Likely | No Trend | No Trend | No Trend |
| Streamflow  Averaging | Total Change in load, kg | 0.229 | 2.88 | 2.01 | 0.145 | 0.573 |
|  | Total Change in yield, kg/km^2^ | 0.0433 | 0.203 | 0.185 | 0.0151 | 0.0819 |
|  | Annual Change, % | 0.974 | 3.07 | 2.47 | 0.453 | 1.24 |
|  | Likelihood of Trend | No Trend | Very Likely | Very Likely | No Trend | No Trend |

Table S19 Trends in flow normalized total dissolved phosphorus loads computed with four different methods. Likelihood of a trend for WRTDS-S and WRTDS is based on the WRTDS bootstrapping test (Hirsch et al., 2015**)** whereas likelihood for LOESS-adjustment and Streamflow-averaging is based on null-hypothesis significance test where 0.05 < p-value ≤ 0.10 is a likely trend and p-value ≤ 0.05 is a very likely trend

| Method | Measure | DEAD | DIFF | FLAT | LONG | SFLIL |
| --- | --- | --- | --- | --- | --- | --- |
| WRTDS-S | Total Change in load, kg | -0.633 | 27.9 | -125 | 0.813 | -7.47 |
|  | Total Change in yield, kg/km^2^ | -0.119 | 1.97 | -11.5 | 0.0847 | -1.07 |
|  | Annual Change, % | -0.0500 | 2.10 | -2.35 | 0.0556 | -0.947 |
|  | Likelihood of Trend | No Trend | No Trend | Very Likely | Very Likely | Very Likely |
| WRTDS | Total Change in load, kg | 14.1 | 126 | -67.0 | -116 | 43.3 |
|  | Total Change in yield, kg/km^2^ | 2.65 | 8.90 | -6.15 | -12.1 | 6.19 |
|  | Annual Change, % | 0.900 | 6.44 | -1.22 | -0.556 | 4.25 |
|  | Likelihood of Trend | Likely | Very Likely | Very Likely | No Trend | Very Likely |
| LOESS  Adjustment | Total Change in load, kg | 1.23 | 3.83 | -12.9 | 2.29 | -0.576 |
|  | Total Change in yield, kg/km^2^ | 0.231 | 0.269 | -1.18 | 0.239 | -0.0823 |
|  | Annual Change, % | 0.834 | 2.23 | -2.94 | 1.52 | -0.860 |
|  | Likelihood of Trend | No Trend | Very Likely | Very Likely | Likely | No Trend |
| Streamflow  Averaging | Total Change in load, kg | 0.110 | 0.822 | -3.68 | -0.0821 | -0.295 |
|  | Total Change in yield, kg/km^2^ | 0.0208 | 0.0579 | -0.338 | -0.00855 | -0.0421 |
|  | Annual Change, % | 0.440 | 1.83 | -2.80 | -0.339 | -1.27 |
|  | Likelihood of Trend | No Trend | Very Likely | Very Likely | No Trend | Very Likely |

Table S20 Trends in flow normalized orthophosphate loads computed with four different methods. Likelihood of a trend for WRTDS-S and WRTDS is based on the WRTDS bootstrapping test (Hirsch et al., 2015**)** whereas likelihood for LOESS-adjustment and Streamflow-averaging is based on null-hypothesis significance test where 0.05 < p-value ≤ 0.10 is a likely trend and p-value ≤ 0.05 is a very likely trend

| Method |  | DEAD | DIFF | FLAT | LONG | SFLIL |
| --- | --- | --- | --- | --- | --- | --- |
| WRTDS-S | Total Change in load, kg | -0.522 | 54.4 | -78.1 | 10.7 | 18.3 |
|  | Total Change in yield, kg/km^2^ | -0.0986 | 3.83 | -7.17 | 1.11 | 2.61 |
|  | Annual Change, % | -0.0429 | 3.73 | -1.60 | 0.922 | 2.93 |
|  | Likelihood of Trend | Likely | Very Likely | No Trend | Likely | Likely |
| WRTDS | Total Change in load, kg | 15.3 | 21.8 | -39.4 | 12.5 | 51.6 |
|  | Total Change in yield, kg/km^2^ | 2.88 | 1.54 | -3.61 | 1.31 | 7.37 |
|  | Annual Change, % | 1.05 | 0.400 | -0.780 | 0.0556 | 3.31 |
|  | Likelihood of Trend | Likely | No Trend | Likely | No Trend | Very Likely |
| LOESS  Adjustment | Total Change in load, kg | 0.501 | 5.35 | -7.92 | 2.81 | 2.01 |
|  | Total Change in yield, kg/km^2^ | 0.0945 | 0.377 | -0.727 | 0.293 | 0.287 |
|  | Annual Change, % | 0.370 | 3.47 | -1.82 | 2.24 | 2.58 |
|  | Likelihood of Trend | No Trend | Very Likely | Very Likely | Very Likely | Very Likely |
| Streamflow  Averaging | Total Change in load, kg | 0.129 | 1.94 | -2.60 | 0.113 | 0.697 |
|  | Total Change in yield, kg/km^2^ | 0.0243 | 0.136 | -0.239 | 0.0118 | 0.0996 |
|  | Annual Change, % | 0.526 | 3.43 | -1.99 | 0.489 | 2.50 |
|  | Likelihood of Trend | No Trend | Very Likely | Very Likely | No Trend | Very Likely |

Table S21 Trends in flow normalized total nitrogen loads computed with four different methods. Likelihood of a trend for WRTDS-S and WRTDS is based on the WRTDS bootstrapping test (Hirsch et al., 2015**)** whereas likelihood for LOESS-adjustment and Streamflow-averaging is based on null-hypothesis significance test where 0.05 < p-value ≤ 0.10 is a likely trend and p-value ≤ 0.05 is a very likely trend

| Method |  | DEAD | DIFF | FLAT | LONG | SFLIL |
| --- | --- | --- | --- | --- | --- | --- |
| WRTDS-S | Total Change in load, kg | 277 | 3480 | -3110 | 2610 | 1860 |
|  | Total Change in yield, kg/km^2^ | 52.2 | 245 | -286 | 272 | 266 |
|  | Annual Change, % | 0.379 | 2.14 | -1.65 | 5.16 | 1.59 |
|  | Likelihood of Trend | Very Likely | Very Likely | Very Likely | Very Likely | Very Likely |
| WRTDS | Total Change in load, kg | -632 | 1490 | -3120 | 756 | 1460 |
|  | Total Change in yield, kg/km^2^ | -119 | 105 | -286 | 78.7 | 209 |
|  | Annual Change, % | -0.821 | 0.740 | -1.59 | 0.944 | 1.16 |
|  | Likelihood of Trend | Very Likely | Likely | Very Likely | Likely | Very Likely |
| LOESS  Adjustment | Total Change in load, kg | -6.65 | 268 | -250 | 312 | 130 |
|  | Total Change in yield, kg/km^2^ | -1.25 | 18.8 | -22.9 | 32.4 | 18.5 |
|  | Annual Change, % | -0.105 | 1.68 | -1.72 | 4.74 | 1.18 |
|  | Likelihood of Trend | No Trend | Very Likely | Very Likely | Very Likely | Very Likely |
| Streamflow  Averaging | Total Change in load, kg | 2.29 | 108 | -84.0 | 56.6 | 113 |
|  | Total Change in yield, kg/km^2^ | 0.433 | 7.57 | -7.71 | 5.90 | 16.1 |
|  | Annual Change, % | 0.0937 | 1.55 | -1.66 | 4.73 | 1.77 |
|  | Likelihood of Trend | No Trend | Very Likely | Very Likely | Very Likely | Very Likely |

Table S22 Trends in flow normalized total particulate nitrogen loads computed with four different methods. Likelihood of a trend for WRTDS-S and WRTDS is based on the WRTDS bootstrapping test (Hirsch et al., 2015**)** whereas likelihood for LOESS-adjustment and Streamflow-averaging is based on null-hypothesis significance test where 0.05 < p-value ≤ 0.10 is a likely trend and p-value ≤ 0.05 is a very likely trend

| Method |  | DEAD | DIFF | FLAT | LONG | SFLIL |
| --- | --- | --- | --- | --- | --- | --- |
| WRTDS-S | Total Change in load, kg | -51.8 | 398 | -495 | 416 | 17.3 |
|  | Total Change in yield, kg/km^2^ | -9.78 | 28.1 | -45.4 | 43.3 | 2.46 |
|  | Annual Change, % | -0.450 | 1.37 | -1.70 | 3.19 | 0.133 |
|  | Likelihood of Trend | Likely | Likely | Very Likely | Very Likely | No Trend |
| WRTDS | Total Change in load, kg | -454 | -342 | -1100 | -161000 | -1010 |
|  | Total Change in yield, kg/km^2^ | -85.7 | -24.1 | -101 | -16800 | -144 |
|  | Annual Change, % | -1.71 | -0.500 | -1.81 | -0.233 | -2.36 |
|  | Likelihood of Trend | Likely | Likely | Very Likely | No Trend | Very Likely |
| LOESS  Adjustment | Total Change in load, kg | -16.1 | 62.3 | -37.4 | 81.2 | 4.80 |
|  | Total Change in yield, kg/km^2^ | -3.03 | 4.39 | -3.43 | 8.46 | 0.686 |
|  | Annual Change, % | -0.787 | 1.33 | -1.11 | 2.89 | 0.257 |
|  | Likelihood of Trend | No Trend | No Trend | No Trend | No Trend | No Trend |
| Streamflow  Averaging | Total Change in load, kg | 0.332 | -5.22 | -1.68 | -0.928 | -0.766 |
|  | Total Change in yield, kg/km^2^ | 0.0626 | -0.368 | -0.154 | -0.0966 | -0.109 |
|  | Annual Change, % | 0.200 | -0.796 | -0.379 | -0.731 | -0.264 |
|  | Likelihood of Trend | No Trend | Likely | Likely | No Trend | No Trend |

Table S23 Trends in flow normalized total dissolved nitrogen loads computed with four different methods. Likelihood of a trend for WRTDS-S and WRTDS is based on the WRTDS bootstrapping test (Hirsch et al., 2015**)** whereas likelihood for LOESS-adjustment and Streamflow-averaging is based on null-hypothesis significance test where 0.05 < p-value ≤ 0.10 is a likely trend and p-value ≤ 0.05 is a very likely trend

| Method |  | DEAD | DIFF | FLAT | LONG | SFLIL |
| --- | --- | --- | --- | --- | --- | --- |
| WRTDS-S | Total Change in load, kg | 337 | 3200 | -1580 | 1880 | 2790 |
|  | Total Change in yield, kg/km^2^ | 63.7 | 225 | -145 | 196 | 399 |
|  | Annual Change, % | 0.529 | 2.31 | -1.08 | 4.97 | 2.69 |
|  | Likelihood of Trend | Very Likely | Very Likely | Very Likely | Very Likely | Very Likely |
| WRTDS | Total Change in load, kg | -245 | 2120 | -1620 | -872 | 2630 |
|  | Total Change in yield, kg/km^2^ | -46.3 | 149 | -149 | -90.8 | 376 |
|  | Annual Change, % | -0.457 | 1.54 | -1.17 | -1.78 | 3.03 |
|  | Likelihood of Trend | Likely | Very Likely | Very Likely | Likely | Very Likely |
| LOESS  Adjustment | Total Change in load, kg | -2.51 | 231 | -130 | 112 | 200 |
|  | Total Change in yield, kg/km^2^ | -0.474 | 16.3 | -11.9 | 11.6 | 28.6 |
|  | Annual Change, % | -0.0554 | 1.85 | -1.21 | 3.06 | 2.06 |
|  | Likelihood of Trend | No Trend | Very Likely | Very Likely | Very Likely | Very Likely |
| Streamflow  Averaging | Total Change in load, kg | 6.95 | 128 | -56.7 | 63.0 | 169 |
|  | Total Change in yield, kg/km^2^ | 1.31 | 9.01 | -5.20 | 6.56 | 24.1 |
|  | Annual Change, % | 0.297 | 1.92 | -1.19 | 5.47 | 2.71 |
|  | Likelihood of Trend | Likely | Very Likely | Very Likely | Very Likely | Very Likely |

Table S24 Trends in flow normalized nitrate plus nitrite loads computed with four different methods. Likelihood of a trend for WRTDS-S and WRTDS is based on the WRTDS bootstrapping test (Hirsch et al., 2015**)** whereas likelihood for LOESS-adjustment and Streamflow-averaging is based on null-hypothesis significance test where 0.05 < p-value ≤ 0.10 is a likely trend and p-value ≤ 0.05 is a very likely trend

| Method |  | DEAD | DIFF | FLAT | LONG | SFLIL |
| --- | --- | --- | --- | --- | --- | --- |
| WRTDS-S | Total Change in load, kg | -135 | 2390 | -2070 | 1380 | 2910 |
|  | Total Change in yield, kg/km^2^ | -25.5 | 168 | -190 | 144 | 416 |
|  | Annual Change, % | -0.264 | 2.22 | -1.91 | 6.26 | 3.23 |
|  | Likelihood of Trend | Likely | Very Likely | Very Likely | Very Likely | Very Likely |
| WRTDS | Total Change in load, kg | -286 | 1430 | -2030 | 406 | 2530 |
|  | Total Change in yield, kg/km^2^ | -53.9 | 101 | -186 | 42.3 | 361 |
|  | Annual Change, % | -0.729 | 1.47 | -2.06 | 1.84 | 3.62 |
|  | Likelihood of Trend | Very Likely | Very Likely | Very Likely | Very Likely | Very Likely |
| LOESS  Adjustment | Total Change in load, kg | -34.0 | 135 | -178 | 60.7 | 195 |
|  | Total Change in yield, kg/km^2^ | -6.42 | 9.50 | -16.3 | 6.33 | 27.9 |
|  | Annual Change, % | -1.07 | 1.55 | -2.53 | 3.07 | 2.28 |
|  | Likelihood of Trend | Very Likely | Very Likely | Very Likely | Very Likely | Very Likely |
| Streamflow  Averaging | Total Change in load, kg | -14.6 | 104 | -81.0 | 55.7 | 193 |
|  | Total Change in yield, kg/km^2^ | -2.75 | 7.32 | -7.43 | 5.80 | 27.6 |
|  | Annual Change, % | -0.719 | 1.93 | -2.29 | 7.11 | 3.25 |
|  | Likelihood of Trend | Very Likely | Very Likely | Very Likely | Very Likely | Very Likely |

Table S25 Trends in flow normalized total Kjeldahl nitrogen loads computed with four different methods. Likelihood of a trend for WRTDS-S and WRTDS is based on the WRTDS bootstrapping test (Hirsch et al., 2015**)** whereas likelihood for LOESS-adjustment and Streamflow-averaging is based on null-hypothesis significance test where 0.05 < p-value ≤ 0.10 is a likely trend and p-value ≤ 0.05 is a very likely trend

| Method |  | DEAD | DIFF | FLAT | LONG | SFLIL |
| --- | --- | --- | --- | --- | --- | --- |
| WRTDS-S | Total Change in load, kg | -19.1 | 998 | -677 | 1400 | -118 |
|  | Total Change in yield, kg/km^2^ | -3.61 | 70.3 | -62.1 | 146 | -16.9 |
|  | Annual Change, % | -0.0857 | 1.65 | -1.01 | 5.84 | -0.473 |
|  | Likelihood of Trend | Likely | Very Likely | Very Likely | Very Likely | No Trend |
| WRTDS | Total Change in load, kg | -317 | 100 | -892 | -32.8 | -1050 |
|  | Total Change in yield, kg/km^2^ | -59.9 | 7.05 | -81.8 | -3.42 | -150 |
|  | Annual Change, % | -0.836 | 0.100 | -0.920 | -0.0556 | -1.73 |
|  | Likelihood of Trend | Likely | No Trend | Likely | No Trend | Very Likely |
| LOESS  Adjustment | Total Change in load, kg | -7.34 | 121 | -33.9 | 262 | -2.84 |
|  | Total Change in yield, kg/km^2^ | -1.38 | 8.54 | -3.11 | 27.3 | -0.406 |
|  | Annual Change, % | -0.238 | 1.49 | -0.513 | 5.89 | -0.101 |
|  | Likelihood of Trend | No Trend | Very Likely | No Trend | Very Likely | No Trend |
| Streamflow  Averaging | Total Change in load, kg | 1.30 | 9.68 | -5.63 | 16.1 | -7.08 |
|  | Total Change in yield, kg/km^2^ | 0.245 | 0.682 | -0.516 | 1.68 | -1.01 |
|  | Annual Change, % | 0.330 | 0.579 | -0.393 | 3.81 | -1.18 |
|  | Likelihood of Trend | No Trend | No Trend | Likely | Very Likely | Very Likely |

Table S26 Trends in flow normalized dissolved total Kjeldahl nitrogen loads computed with four different methods. Likelihood of a trend for WRTDS-S and WRTDS is based on the WRTDS bootstrapping test (Hirsch et al., 2015**)** whereas likelihood for LOESS-adjustment and Streamflow-averaging is based on null-hypothesis significance test where 0.05 < p-value ≤ 0.10 is a likely trend and p-value ≤ 0.05 is a very likely trend

| Method |  | DEAD | DIFF | FLAT | LONG | SFLIL |
| --- | --- | --- | --- | --- | --- | --- |
| WRTDS-S | Total Change in load, kg | 184 | 877 | -132 | 683 | 156 |
|  | Total Change in yield, kg/km^2^ | 34.7 | 61.8 | -12.1 | 71.1 | 22.3 |
|  | Annual Change, % | 1.44 | 2.30 | -0.313 | 4.62 | 1.29 |
|  | Likelihood of Trend | Very Likely | Very Likely | Very Likely | Very Likely | Very Likely |
| WRTDS | Total Change in load, kg | 112 | 886 | 452 | 232 | 256 |
|  | Total Change in yield, kg/km^2^ | 21.2 | 62.4 | 41.5 | 24.1 | 36.5 |
|  | Annual Change, % | 0.807 | 2.30 | 1.13 | 1.08 | 1.49 |
|  | Likelihood of Trend | Very Likely | Very Likely | Very Likely | Likely | Very Likely |
| LOESS  Adjustment | Total Change in load, kg | 18.9 | 81.2 | -5.38 | 58.9 | 19.8 |
|  | Total Change in yield, kg/km^2^ | 3.57 | 5.72 | -0.494 | 6.14 | 2.83 |
|  | Annual Change, % | 1.47 | 2.05 | -0.147 | 3.68 | 1.61 |
|  | Likelihood of Trend | Very Likely | Very Likely | No Trend | Very Likely | Very Likely |
| Streamflow  Averaging | Total Change in load, kg | 4.68 | 27.7 | -5.06 | 14.9 | 3.35 |
|  | Total Change in yield, kg/km^2^ | 0.883 | 1.95 | -0.464 | 1.55 | 0.48 |
|  | Annual Change, % | 1.49 | 2.04 | -0.423 | 4.08 | 0.74 |
|  | Likelihood of Trend | Very Likely | Very Likely | Very Likely | Very Likely | Very Likely |


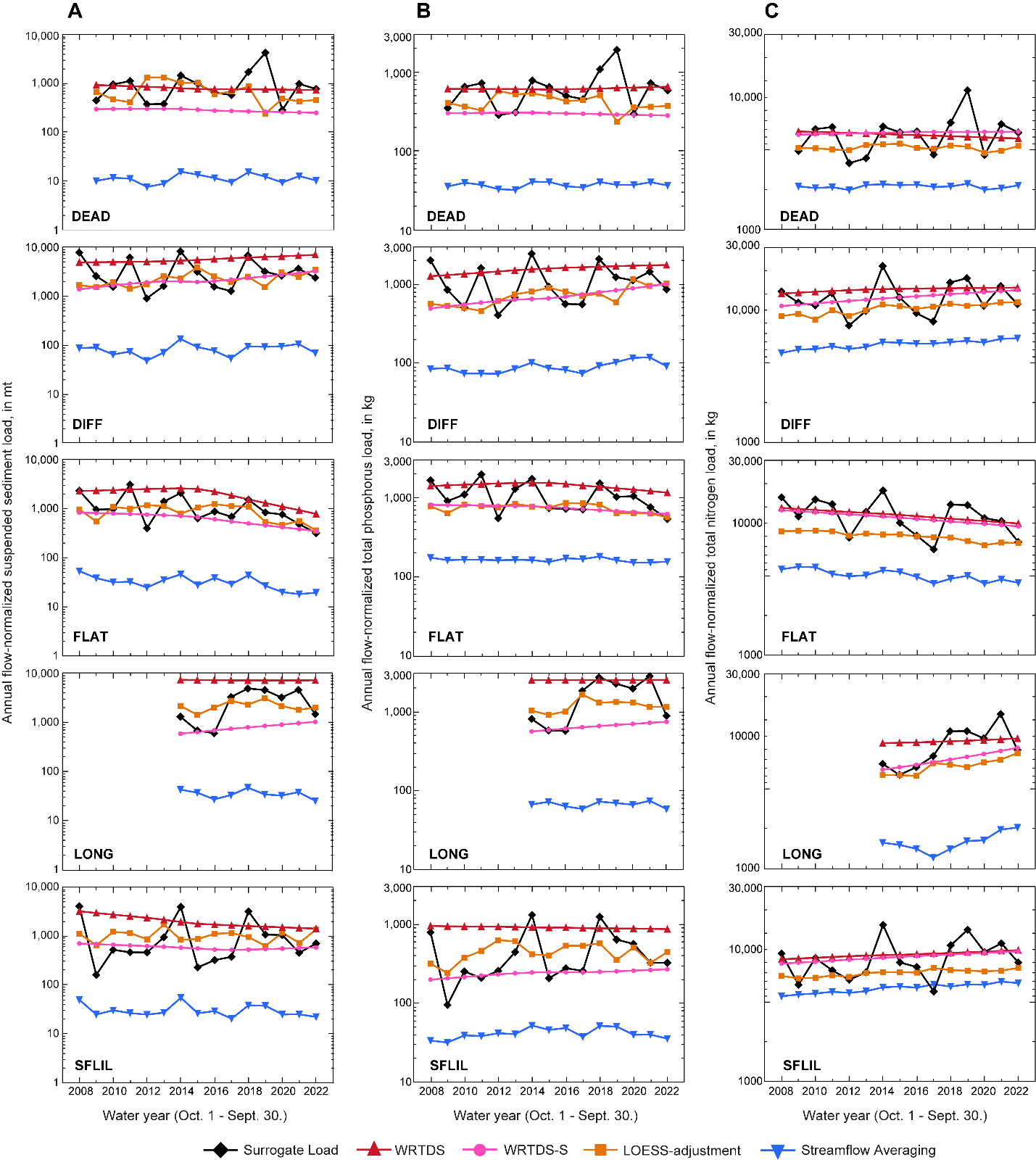


Fig S1 1 Annual A) suspended sediment, B) total phosphorus, and C) total nitrogen loads computed with surrogate regression models and annual flow-normalized loads computed with four different methods at the five intensively monitored watersheds

References

American Public Health Association (APHA), American Water Works Association (AWWA), & Water Environment Federation (WEF). (2017). *Standard methods for the examination of water and wastewater* (23rd ed.). Washington, DC: American Public Health Association.

ASTM International. (2019). *ASTM D3977-97(2019) Standard test methods for determining sediment concentration in water samples.* ASTM International. https://doi.org/10.1520/D3977-97R19

Cleveland, W. S., & Devlin, S. J. (1988). Locally weighted regression: An approach to regression analysis by local fitting. *Journal of the American Statistical Association, 83*(403), 596–610. <https://doi.org/10.2307/2289282>

Fairfax County (2023). 2023 Municipal Separate Storm Sewer System (MS4) Program Plan and Annual Report. Accessed August 20, 2024 at <https://www.fairfaxcounty.gov/publicworks/sites/publicworks/files/Assets/Documents/PDF/reports/MS4/2023-ms4-plan-report.pdf>

Fairfax County (2024). Fairfax County, Virginia: FY 2024 – FY 2028 Adopted CIP - 223, Retrieved March 30, 2024, from <https://www.fairfaxcounty.gov/budget/sites/budget/files/Assets/documents/fy2024/adopted/cip/13-STORMWATER%20MANAGEMENT.pdf>

Hirsch, R.M., Archfield, S.A., & De Cicco, L.A. (2015). A bootstrap method for estimating uncertainty of water quality trends. *Environmental Modelling & Software*, 73, 148-166, <https://doi.org/10.1016/j.envsoft.2015.07.017>

U.S. Environmental Protection Agency (EPA). (1974). *Method 365.2: Determination of phosphorus by semi-automated colorimetry.* U.S. Environmental Protection Agency, Office of Research and Development.

U.S. Environmental Protection Agency (EPA). (1978). *Method 351.2: Determination of total Kjeldahl nitrogen by semi-automated colorimetry.* U.S. Environmental Protection Agency, Office of Research and Development.

U.S. Environmental Protection Agency (EPA). (1978). *Method 353.2: Determination of nitrate-nitrite by automated colorimetry.* U.S. Environmental Protection Agency, Office of Research and Development.

U.S. Environmental Protection Agency (EPA). (1978). *Method 351.2: Determination of total Kjeldahl nitrogen by semi-automated colorimetry.* U.S. Environmental Protection Agency, Office of Research and Development.

U.S. Environmental Protection Agency (EPA). (2010). *Chesapeake Bay Total Maximum Daily Load for Nitrogen, Phosphorus, and Sediment.* U.S. Environmental Protection Agency, Chesapeake Bay Program Office. Available at: <https://www.epa.gov/chesapeake-bay-tmdl>

Webber, J.S., Chanat, J.G., Porter, A.J., & Jastram, J.D. (2022). Climate, landscape, and water-quality metrics for selected watersheds in Fairfax County, Virginia, 2007–2018: *U.S. Geological Survey data release*. <https://doi.org/10.5066/P9FW7KLH>
